# Consensus Paper: Cerebellar Development

**DOI:** 10.1007/s12311-015-0724-2

**Published:** 2015-10-06

**Authors:** Ketty Leto, Marife Arancillo, Esther B. E. Becker, Annalisa Buffo, Chin Chiang, Baojin Ding, William B. Dobyns, Isabelle Dusart, Parthiv Haldipur, Mary E. Hatten, Mikio Hoshino, Alexandra L. Joyner, Masanobu Kano, Daniel L. Kilpatrick, Noriyuki Koibuchi, Silvia Marino, Salvador Martinez, Kathleen J. Millen, Thomas O. Millner, Takaki Miyata, Elena Parmigiani, Karl Schilling, Gabriella Sekerková, Roy V. Sillitoe, Constantino Sotelo, Naofumi Uesaka, Annika Wefers, Richard J. T. Wingate, Richard Hawkes

**Affiliations:** 1 Department of Neuroscience Rita Levi Montalcini, University of Turin, via Cherasco 15, 10026 Turin, Italy; 2 Neuroscience Institute Cavalieri-Ottolenghi, University of Turin, Regione Gonzole 10, 10043 Orbassano, Torino Italy; 3 Departments of Pathology & Immunology and Neuroscience, Baylor College of Medicine, Jan and Dan Duncan Neurological Research Institute of Texas Children’s Hospital, 1250 Moursund Street, Suite 1325, Houston, TX 77030 USA; 4 Medical Research Council Functional Genomics Unit, Department of Physiology, Anatomy and Genetics, University of Oxford, Oxford, OX1 3PT UK; 5 Department of Cell and Developmental Biology, Vanderbilt University Medical Center, 4114 MRB III, Nashville, TN 37232 USA; 6 Department of Microbiology and Physiological Systems and Program in Neuroscience, University of Massachusetts Medical School, 368 Plantation Street, Worcester, MA 01605-2324 USA; 7 Seattle Children’s Research Institute, Center for Integrative Brain Research, Seattle, WA USA; 8 Department of Pediatrics, Genetics Division, University of Washington, Seattle, WA USA; 9 Sorbonne Universités, Université Pierre et Marie Curie Univ Paris 06, Institut de Biologie Paris Seine, France, 75005 Paris, France; 10 Centre National de la Recherche Scientifique, CNRS, UMR8246, INSERM U1130, Neuroscience Paris Seine, France, 75005 Paris, France; 11 Laboratory of Developmental Neurobiology, The Rockefeller University, New York, NY 10065 USA; 12 Department of Biochemistry and Cellular Biology, National Institute of Neuroscience, National Center of Neurology and Psychiatry, 4-1-1 Ogawa-Higashi, Kodaira, Tokyo, 187-8502 Japan; 13 Developmental Biology Program, Sloan Kettering Institute, New York, NY 10065 USA; 14 Department of Neurophysiology, Graduate School of Medicine, The University of Tokyo, Tokyo, 113-0033 Japan; 15 Department of Integrative Physiology, Gunma University Graduate School of Medicine, 3-39-22 Showa-machi, Maebashi, Gunma 371-8511 Japan; 16 Blizard Institute, Barts and The London School of Medicine and Dentistry, Queen Mary University of London, 4 Newark Street, London, E1 2AT UK; 17 Department Human Anatomy, IMIB-Arrixaca, University of Murcia, Murcia, Spain; 18 Department of Anatomy and Cell Biology, Nagoya University Graduate School of Medicine, Nagoya, Japan; 19 Anatomie und Zellbiologie, Anatomisches Institut, Rheinische Friedrich-Wilhelms-Universität, Bonn, Germany; 20 Department of Physiology, Feinberg School of Medicine, Northwestern University, Chicago, IL 60611 USA; 21 Institut de la Vision, UPMC Université de Paris 06, Paris, 75012 France; 22 Center for Neuropathology, Ludwig-Maximilians-University, Munich, Germany; 23 MRC Centre for Developmental Neurobiology, King’s College London, London, UK; 24 Department of Cell Biology & Anatomy and Hotchkiss Brain Institute, Cumming School of Medicine, University of Calgary, Calgary, T2N 4NI AB Canada

**Keywords:** Cerebellum, Progenitors, Purkinje cells, Specification, Differentiation

## Abstract

The development of the mammalian cerebellum is orchestrated by both cell-autonomous programs and inductive environmental influences. Here, we describe the main processes of cerebellar ontogenesis, highlighting the neurogenic strategies used by developing progenitors, the genetic programs involved in cell fate specification, the progressive changes of structural organization, and some of the better-known abnormalities associated with developmental disorders of the cerebellum.

## Introduction (C. Sotelo)

The work done on cerebellar development from the late nineteenth century until the 1970s provided substantial and significant information; however, it was only descriptive and barely addressed the mechanisms involved. Over the last two decades, thanks to the technological revolution in molecular biology, our understanding of cerebellar development has drastically changed. We are now going through an exceptional period in our understanding of the mechanisms that underlie the complex development of the cerebellum. An understanding of cell specification regulated by the expression of region-specific combinations of transcription factors or proneural genes, and the formation of synaptic circuits, seems within reach.

Ferdinando Rossi, a few months before his death, undertook the monumental task of writing a monograph on the spectacular advances in our understanding of cerebellar development achieved in the last 20 years. Sadly, Ferdinando died a few months after beginning his monograph. This consensus paper, based on Ferdinando’s initial design, summarizes many of these advances and is dedicated to his memory.

The review comprises 18 brief sections, ranging from the early molecular specification of the cerebellar anlage to its mature architecture and pathology. It also includes information on neurogenesis, mainly the specification and origins of neuronal and glial progenitors. An important part of the paper is devoted to Purkinje cells (PCs) as key neurons of the cerebellar cortex responsible for the proliferation of granule cells (GCs) and the establishment of “crude” projection maps with extracerebellar afferent fibers. Finally, the biochemical heterogeneity of PCs allows for a cortical subdivision into distinct functional bands, a presumptive protomap for the development of circuit topography (see in [1]). In this context, the problem of synapse elimination in the process of refinement and stabilization of climbing fiber (CF) connections is also summarized.

### The Molecular Specification of the Cerebellar Anlage: The Isthmic Organizer (S. Martinez)

The description of morphogenetic regulatory processes at specific locations of the developing neural primordium has led to the concept of secondary organizers, which regulate the identity and regional polarity of neighboring neuroepithelial regions [2]. These organizers usually develop within the previously broadly regionalized neuroectoderm with defined genetic boundaries. Their subsequent activity refines local neural identities along the anteroposterior or dorsoventral axes, thus regionalizing the anterior neural plate and neural tube [3, 4].

**Fig. 1:**
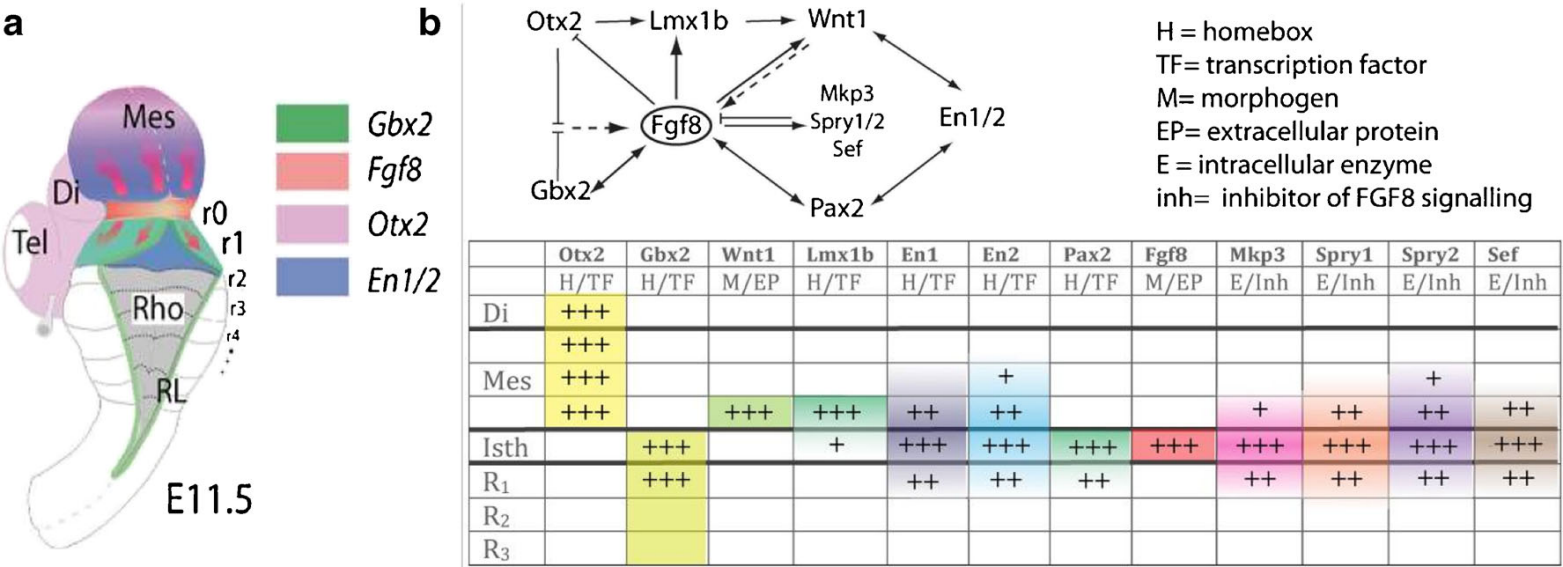
Topographical location of the mid-hindbrain boundary in the E11.5 mouse embryo. **a** Dorsal view of an E11.5 mouse embryo illustrating the isthmic constriction (isth) located between the mesencephalon and rhombomere 1 (r1). Rhombomeres r0 and r1, which give rise to the cerebellum, are highlighted in (**a**). The different color codes depict the expression pattern of the most important genes related to the morphogenetic activity and the capacity of the IsO. **b** Functional interactions (induction/inhibition) of genes that, together with Fgf8, are involved in the molecular maintenance of the isthmic region at E9.5. The table summarizes the expression intensity and expression range of genes along the AP axis of the neural tube, focusing on the isthmus: the level of RNAm expression for each gene is represented by the number of (+) and the *color* signifies the region of expression and the expression pattern (homogeneous or gradient), extending rostrally or caudally from the isthmus. *Tel* telencephalon, *Di* diencephalon, *Mes* mesencephalon. Modified from [4]

The isthmic constriction of the neural tube contains the isthmic organizer (IsO; Fig. 1a), which provides structural polarity to the adjoining regions and orchestrates the complex cellular diversity of the mesencephalon (rostrally) and the cerebellum (caudally; [5, 6]; for reviews see [4, 7]). Cerebellar development is dependent on IsO signaling [5]. The molecular nature of the signal has been identified as a member of the fibroblast growth factor (FGF) family, FGF8, which is highly expressed in the most anterior hindbrain. Indeed, beads containing FGF8 protein mimic the activity of the IsO tissue when ectopically transplanted [8] (Fig. 1a).

The earliest molecular event in IsO specification is the differential expression in the neural plate of Otx2 and Gbx2. In the avian embryo at Hamburger and Hamilton (HH) stage 8 [9], an Otx2 and Gbx2 negative neuroepithelial gap separates these domains, but by HH9 they come to overlap across the prospective mid-hindbrain boundary [10]. Then, *Fgf8* expression is activated (at HH9 in chick and at embryonic day (E) 8.5 in mice) at the interface of the OTX2- and GBX2-positive neuroepithelial domains. The co-expression of *Otx2* and *Gbx2* in the IsO territory essentially disappears by HH11–12 (chick) and E10 (mouse), and both domains become thereafter mutually excluded and complementary. The limit determined by Otx2 and Gbx2 marks the mid-hindbrain molecular boundary (MHB) [10–13]. Secondarily, Lmx1b and Wnt1 are co-expressed in a thin band confined to the caudalmost Otx2 expression domain, abutting the Fgf8 domain at the most rostral edge of the hindbrain. Note that although early *Fgf8* expression appears in the territory co-expressing *Otx2*/*Gbx2*, double deletion of these two genes encoding transcription factors does not primarily affect the activation of *Fgf8* expression [13, 14]. Other genes expressed at early stages across the prospective MHB, such as *Pax*-*2* and *Iroquas* (*Irxs*), seem also to be required for the expression of *Otx2*, *Gbx2*, and *Fgf8* and the proper formation of the mesencephalic and rhombencephalic vesicles. Moreover, FGF8 signaling may act at the IsO in concert with other signaling molecules, such as WNT1, sonic hedgehog (SHH), and transforming growth factor (TGF)-β family members. The morphogenetic activity of the IsO is thus the consequence of the specific temporo-spatial expression of molecular signals that regulate the specification and structural development of mesencephalic and cerebellar neuroepithelial territories (Fig. 1b). Alterations of *Fgf8* and *Gbx2* gene expression lead to massive disruption of the mid-hindbrain neural territory due to gene patterning deregulation [15]. A decreasing gradient of FGF8 protein concentration in the alar plate of the isthmus and rhombomere 1 (r1) is fundamental for cell survival and the differential development of cerebellar regions [7, 16, 17].

Finally, in the proposed mechanism by which FGF8 signaling spreads over a field of target cells, at least in zebrafish, patterning is established and maintained by two essential factors: first, the free diffusion of FGF8 molecules away from the secretion source through the extracellular space and secondly an absorptive function of the receiving cells regulated by FGF receptor-mediated endocytosis [18]; reviewed in [4]. The differential orientation and polarity of the FGF8 signal seems to be directly dependent on the spatial position of FGF8-related secondary organizers and on the activity of the negative modulators MKP3, SEF, and sprouty1/2 (SPRY1/2). FGF8 may also translocate into the nucleus, and this nuclear FGF8 may function as a transcriptional regulator to induce *Spry2* in the isthmus independently of ERK phosphorylation. Similar findings in mouse showed that maintenance of the *Spry2* expression pattern along the isthmic region occurs in the absence of both FGF8 in the extracellular compartment and ERK phosphorylation (reviewed in [4]).

At E9, following territorial specification and the closure of the neural tube, murine cerebellar histogenesis begins with the specification of cerebellar progenitors. Several studies have demonstrated that all cerebellar cells are generated by the neuroepithelium of the alar plate of r1 [19–22]. Conversely, the most dorsal region of r1 gives rise to the roof plate, which produces cells of the choroid plexus [23].

## Specification of Cerebellar Progenitors (M. Hoshino)

All cerebellar neurons are produced in the alar plate of r1 that is located rostrally adjacent to the isthmus. In this region, the dorsalmost part of the neuroepithelium gives rise to the roof plate while the ventrally and intermediately located parts become the ventricular zone (VZ) and the rhombic lip (RL).

Recent genetic and viral lineage tracing studies have clarified the origins and birthdates of distinct subtypes of cerebellar neurons. Cerebellar glutamatergic and GABAergic neurons are generated from the RL and the VZ respectively. In mice, glutamatergic neurons in the cerebellar nuclei (CN) leave the cerebellar RL at early stages (E10.5–12.5) and GCs at middle to late stages (E13.5 onward) [24–26]. Unipolar brush cells (UBCs) are known to emerge at relatively late developmental stages [27]. In mice, PCs are born at E10.5–E13.5, GABAergic interneurons (INs) in the CN at E10.5–E11.5, and Golgi cells at approximately E13.5–postnatal (peak around E14–E16) [28–31]. Late-born GABAergic INs, including stellate and basket cells, derive from secondary precursors in the prospective white matter (PWM) at later stages (from E13 to P5 with a peak around birth) [32, 33]. Thus, cerebellar neuronal subtypes depend on when and where they are generated from neural progenitors. This leads to the idea that cerebellar progenitors with their own spatial and temporal identities produce specific neuronal subtypes.

Two basic-helix-loop-helix (bHLH) proteins, ATHO1 (also called MATH1) and PTF1a, participate in the specification of the spatial identities of cerebellar progenitors. *Atoh1* is expressed in the progenitors of the RL, and targeted *Atoh1* disruption results in the loss of glutamatergic neurons in the cerebellum [24–26]. On the other hand, *Ptf1a* is expressed in the VZ progenitors and *Ptf1a* deletion results in the loss of all cerebellar GABAergic neurons [34, 35]. Furthermore, mis-expression of ATHO1 and PTF1a in the VZ and the RL results in the ectopic production of glutamatergic and GABAergic neurons, respectively [36]. These facts suggest that *Atoh1* and *Ptf1a* confer the spatial identities of the RL and the VZ on cerebellar progenitors to produce glutamatergic and GABAergic neurons, respectively.

*Atoh1* expression in the RL is regulated by TGFβ and Delta-Notch signaling [37–41]. *Ptf1a* expression is influenced by SHH signals [42]. PTF1a and ATHO1 can downregulate each other’s expression—forced expression of *Atoh1* suppresses *Ptf1a* and, conversely, forced expression of *Ptf1a* suppresses *Atoh1* [36, 43]. Consistent with this view, *Atoh1* expression ectopically expands into the VZ in *Ptf1a* mutants [35, 36]. However, ectopic *Ptf1a* expression is not observed in the RL of *Atoh1* mutants [36], suggesting that PTF1a suppresses the expression of *Atoh1* but the converse is not physiologically true in the cerebellar primordium.

### Glutamatergic Phenotypes

**Fig. 2:**
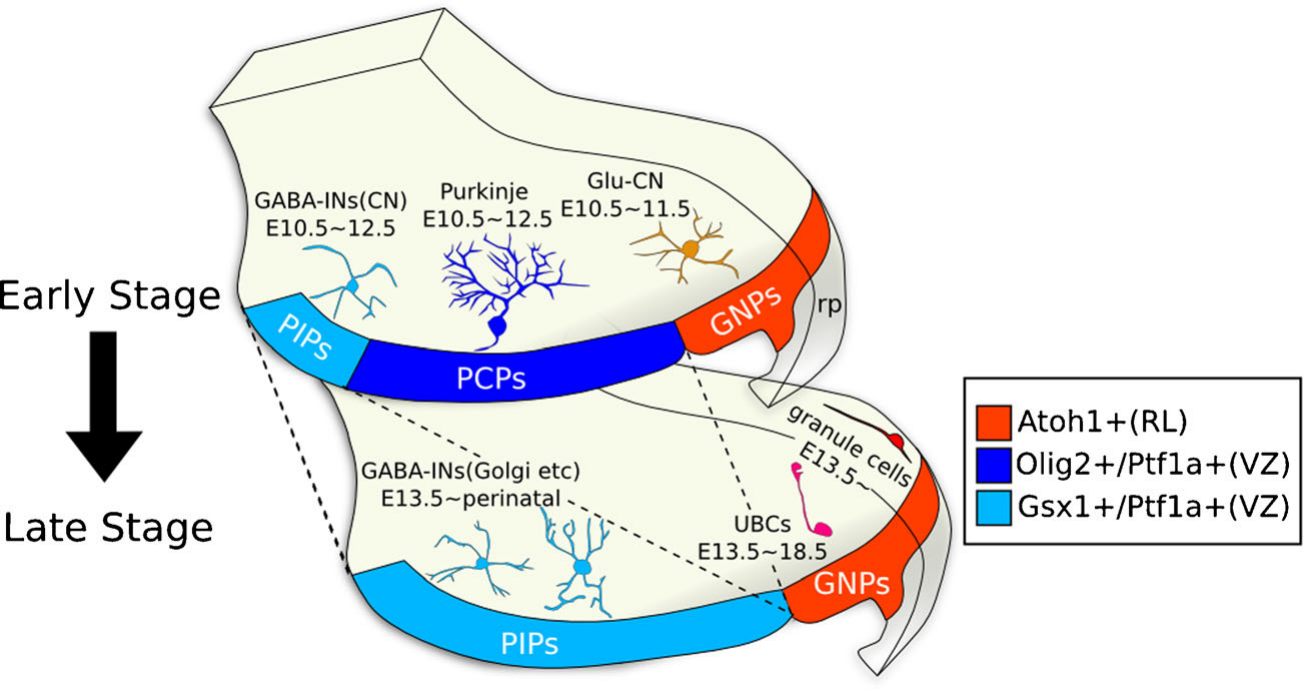
Progenitors and neurons in the cerebellum. Right is dorsal, left is ventral. *GNPs* glutamatergic neuron progenitors, *PIPs* Pax-2^+^ IN progenitors, *PCPs* Purkinje cell progenitors, *rp* roof plate, *GABA-INs (CN)* GABAergic interneurons in the cerebellar nuclei, *Glu-CN* glutamatergic neurons in the cerebellar nuclei

In contrast to the GABAergic neuron progenitors in the VZ, the machinery to specify cerebellar glutamatergic neuron subtypes remains elusive. However, even when glutamatergic neurons are ectopically produced from the VZ by ectopic expression of ATHO1, the generation of neuronal types follows the temporal schedule of the normal glutamatergic neurons derived from the RL [36]. This suggests that, as for GABAergic neuron progenitors, glutamatergic neuron progenitors in the RL may change their temporal identities from “glutamatergic CN neuron-producing type” to “GC/UBC-producing type” during development (Fig. 2). In the RL at late stages, some progenitors express either GC (PAX6) or UBC (TBR2) markers [27]. This suggests that GCs and UBCs may be produced from distinct progenitors in the RL, although some cells in the RL are found to express both markers (see also “Unipolar brush cells” section). Because the loss of the roof plate or targeted disruption of genes expressed in the roof plate affects the morphology and the nature of the RL [23, 44, 45], extrinsic factors from the roof plate may also play important roles to regulate the identities of glutamatergic neuron progenitors in the RL.

### GABAergic Phenotypes

Cerebellar GABAergic neurons can be categorized into two subtypes: PCs and Pax-2^+^ INs. Each subtype is generated from a distinct progenitor in the VZ:PC progenitors (PCPs) and Pax-2^+^ IN progenitors (PIPs). At the early stages, only a small number of PIPs are located in the rostralmost region of the VZ and a large number of PCPs occupy the remaining regions in the VZ. As development proceeds, PCPs gradually transit to become PIPs spreading from ventral to dorsal. This temporal identity transition of cerebellar GABAergic neuron progenitors from PCPs to PIPs is negatively regulated by Olig2 and positively by Gsx1 [46].

The VZ subregion containing PCPs is also characterized by the strong expression of E-cadherin and the cell surface marker Neph3, which is a direct downstream target gene of PTF1a [47, 48]. Moreover, downstream of PTF1a, Neurogenin1 and Neurogenin2 are expressed in the VZ and implicated in cell-cycle control and PC development [49, 50]. Expression of LHX1/LHX5 [23, 51] and Corl2 [52], seen in the subventricular zone, indicates the commitment of VZ-born daughter cells to a PC fate, and these markers are useful for assessing the differentiation of embryonic stem (ES) cell-derived PCs [53].

Based on the birthdates of distinct PAX-2^+^ INs, PIPs may first produce GABAergic INs in the CN (from ~E10.5) and then generate Golgi cells (from ~E13.5; Fig. 2). At later stages of neurogenesis, PIPs may give rise to precursors in the PWM that eventually generate the stellate and basket cells.

### Patterning of the Cerebellar Cortex: Rhombic Lip-Derived Phenotypes

#### Granule Cells

During cerebellar development, granule cell progenitors (GCPs) arise in the RL and undergo a prolonged clonal expansion that generates a population of granule neurons that outnumbers the total neuronal population of the cerebral cortex.

#### Cerebellar Granule Cell Neurogenesis and Migration (M. E. Hatten)

**Fig. 3:**
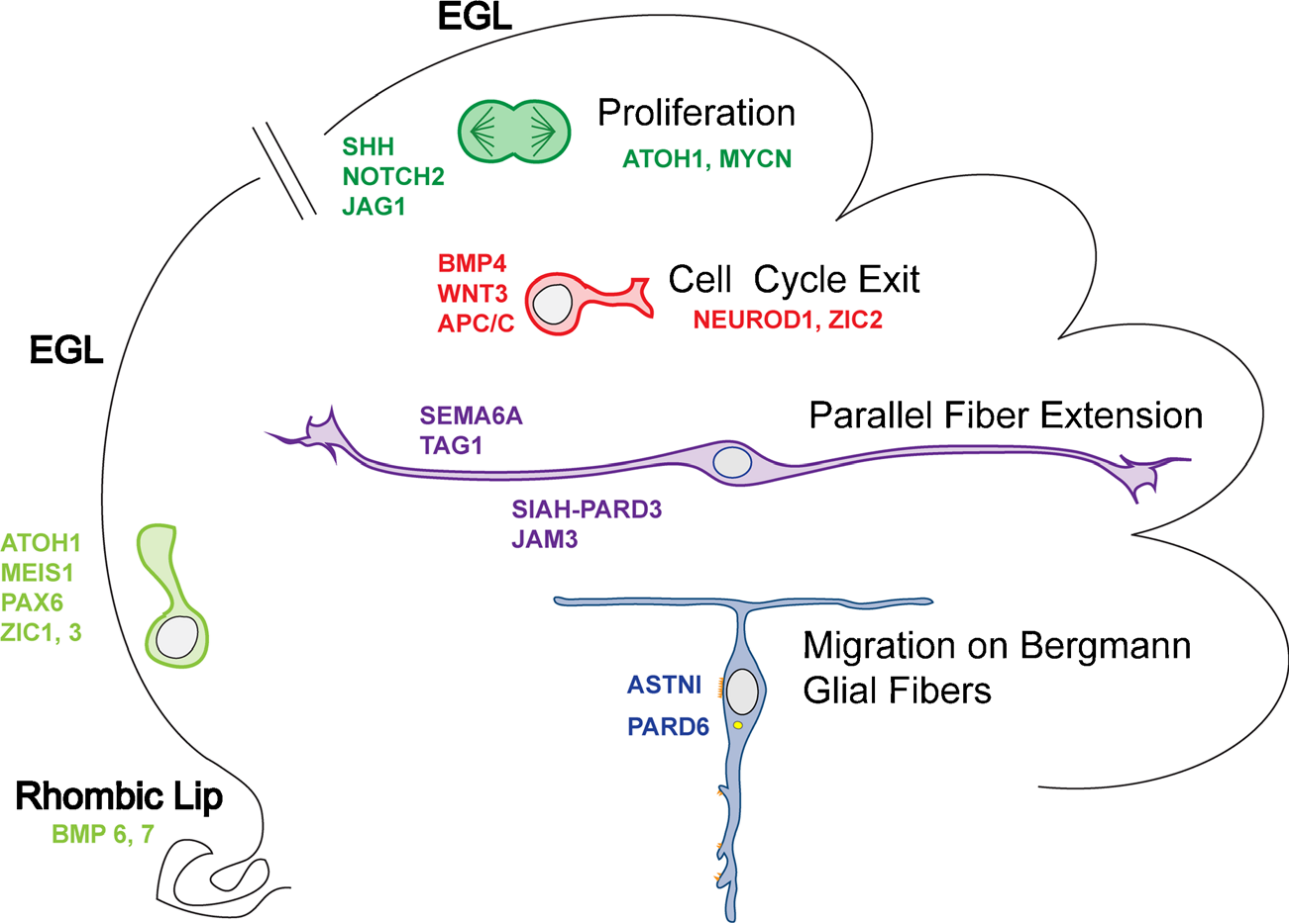
Granule cell neurogenesis and migration. GCPs arise in the RL of the cerebellar anlagen, after which early, proliferating GCPs (*light green*) migrate across the surface of the anlagen to form the EGL. After birth (*broken line*), GCPs located in the outer aspect of the EGL (*dark green*) express the transcription factors (TFs) MATH1/ATOH1 and NMYC, and proliferate in response to the mitogens Shh and JAG1 (NOTCH2). BMP4, WNT3, and APC/C inhibit GCP proliferation and promote cell-cycle exit. Postmitotic GCPs (*red*) express NEUROD1 and ZIC2. SIAH-PARD3 and JAM3, as well as SEMA6A, promote exit from the outer EGL where GCPs extend TAG1 positive PF axons (*purple*). Migrating neurons (*blue*) extend a leading process tipped with short filopodia and lamellipodia that enwraps the glial fiber (not shown). As the neuron moves, a broad interstitial junction (*yellow*) is formed beneath the cell soma that contains the neuron-glial adhesion protein ASTN1. PARD6, localized at the centrosome (*yellow*), coordinates the forward movement of the soma and centrosome through activation of Myosin II-Actin motors that pull the cell forward. *EGL* external granular layer

Between E12.5 and E16, RL-derived precursors spread across the dorsal surface of the cerebellar anlagen to form the external granular layer (EGL) [54]. At this stage, RL-derived progenitors express the bHLH transcription factor gene Atoh1 [25, 26, 55], the zinc finger protein genes Zic1 and Zic3 [56], the homeobox gene *Meis1* [51], the paired box gene 6 *Pax6* [57], and the calmodulin-dependent phosphodiesterase 1C gene (*Pde1c*) [58] (Fig. 3). *Atoh1* expression is induced by BMP signaling in the choroid plexus and the roof plate [55, 59] and maintained by the roof plate organizer [41]. While the vast majority of ATOH1^+^ RL derivatives generate GCPs that migrate tangentially in the EGL, fate mapping experiments indicate that a subpopulation migrates rostrally to the nascent CN [25, 26, 51, 60] (see “Development of the Cerebellar Nuclei” section below). Recent studies demonstrate that FGF signaling allocates discrete subpopulations of RL-derived ATOH1^+^ cells, with downregulation of FGF signaling being required to generate RL-derived cerebellar neurons [61].

During the early postnatal period, multiple mitogenic pathways expand the EGL from a thin layer to a layer six to eight cells deep. SHH provided by PC neurons is a major driver of GCP proliferation [62]. Molecular genetic studies demonstrate that ATHO1 [24] and MYCN [63] are required for GC specification and the expansion of the pool of GCPs in the early postnatal period. The importance of *Shh* to cerebellar histogenesis is underscored by elegant genetic analyses demonstrating that levels of SHH signaling control the foliation patterning of the cerebellar cortex [64] (see “Cerebellar Foliation” section), and studies on human medulloblastoma (MB) implicate defects in SHH signaling in MB formation (reviewed in [65], see “Deregulated Developmental Pathways in Medulloblastoma” section). The Notch2 pathway also stimulates the expansion of GCPs during cerebellar development as treatment of GCPs with jagged 1 (JAG1), a ligand of Notch2, markedly stimulates GCP proliferation and inhibits GC differentiation [66]. Genetic studies demonstrate that one mechanism for the action of activated NOTCH2 involves antagonizing BMP signaling [67] and upregulating *Atoh1* expression [40]. Support for these key steps in GCP neurogenesis comes from the differentiation of mES cells into GCPs by stepwise treatment with morphogens that establish the cerebellar territory (WNT1, FGF8) [68], followed by BMPs that specify a GCP identity [55]. Subsequent treatment with SHH and JAG1 expands the ES-derived GCPs and exposure to BDNF supports terminal differentiation [68]. Recent gene expression studies using ES cells that express a NeuroD1 translating ribosome affinity purification (TRAP) tag to facilitate purification of GCP RNA show that the transcriptome of ES-derived GCPs generated by this approach approximates that of P7 GCs (Zhu, Tamayo, Mesirov, and Hatten, unpublished results).

Following clonal expansion in the EGL, GCPs exit the cell cycle, downregulating ATOH1 and upregulating NeuroD1 [69], which is required for GCP differentiation [70]. Several pathways are thought to provide negative regulation of GCP proliferation, including BMP4, WNT3, and the anaphase-promoting complex/cyclosome (APC/C^Cdh1^) ubiquitin ligase. WNT3 suppresses GCP growth through a non-canonical WNT signaling pathway, activating prototypic mitogen-activated protein kinases (MAPKs), the RAS-dependent extracellular-signal-regulated kinases 1/2 (ERK1/2) and ERK5, instead of the classical β-catenin pathway [71]. WNT3 inhibits GCP proliferation by downregulating proliferative target genes of the mitogen SHH and the bHLH transcription factor ATHO1 [71]. CK1δ is another novel regulator of GCP expansion as a loss of *Ck1δ* or treatment of GCPs with a highly selective small molecule CK1δ inhibitor inhibits GCP expansion. CK1δ is targeted for proteolysis via APC/C^Cdh1^ ubiquitin ligase, and conditional deletion of the APC/C^Cdh1^ activator, *Cdh1*, in cerebellar GCPs results in higher levels of CK1δ, suggesting an important role for the APC/C^Cdh1^ complex in GCP cell cycle exit [72].

Postmitotic GCPs express the axonal glycoprotein TAG1 (CNT2), a contactin-related adhesion molecule [73, 74], which functions in parallel fiber (PF) extension. Genetic experiments show Semaphorin 6A (Sema6A) functions in the switch from tangential migration in the EGL to radial migration along Bergmann glia (BG) [75] by a mechanism that involves binding to Plexin A2 [76]. Recent live imaging and functional studies also demonstrate a critical role for the SIAH E3 ubiquitin ligase, which controls proteosomal degradation the Pard3A polarity protein and regulates GCP adhesion during EGL exit via the junctional adhesion molecule JAM-C [77]. During PF extension, the cell soma extends a leading process along the radial BG fiber [78]. Live imaging experiments demonstrate that migrating GCPs form an extensive adhesion junction beneath the cell surface involving the neuron-glial adhesion protein ASTN1 [79–82] and extend a leading process with short filopodia and lamellipodia that enwrap the glial fiber [83, 84]. Forward movement of the cell soma follows the release of the neuron-glial adhesion junction beneath the cell body, after which the neuron glides along the glial fiber until a new adhesion forms [84]. Live imaging functional studies show that the PAR6 polarity complex localizes to the centrosome and coordinates the forward movement of the centrosome and soma [85] by a mechanism that includes activation of actomyosin contractile motors in the proximal region of the leading process [86]. These studies suggest a model by which actomyosin contractility in the leading process, rather than in a classical “leading edge” at the tip of the leading process, provides the force needed for forward movement. Postmigratory GCs settle in the nascent granular layer (GL) where they extend dendrites and form synapses with mossy fiber afferent axons (inter alia).

#### Voltage-Sensitive Regulation of Dendrite Formation and its Timing in Granule Cells (B. Ding and D. L. Kilpatrick)

GCs relay and process neural inputs into the cerebellum from mossy fiber afferents to PC efferents. Each GC dendrite apparently synapses with a single mossy fiber, which has the capacity to promote combinatorial encoding and the enhanced processing of sensory input to the cerebellum [87]. The developmental regulation of GC dendrite/synapse formation is therefore central to cerebellar circuitry.

Studies of dendrite-related timing mechanisms in GCs initially focused on the *Gabra6* gene encoding the GABA_A_ receptor α6 subunit, which exhibits delayed or “late” gene expression in maturing GCs [88]. These and related studies revealed that the Nuclear Factor One (NFI) transcription factor family, and in particular NFIA, is important for dendrite formation and *Gabra6* expression in maturing GCs [88–90]. Further, while NFI proteins are constitutively expressed in the nucleus throughout GC maturation, the occupancy of an essential NFI binding site within the *Gabra6* promoter closely mirrors the temporal onset of *Gabra6* expression [88]. These studies directly implicated NFI occupancy as a timing mechanism for *Gabra6* expression.

What regulates NFI temporal binding to the *Gabra6* gene in developing GCs? Wang et al. [88] found that NFI occupancy in pre-migratory GCs, prior to departure from the EGL and arrival in the GL, is inhibited by binding of the *trans*-repressor REST to the *Gabra6* promoter. REST expression and DNA binding decline as GCPs initiate differentiation, and knockdown of REST selectively enhances the onset of *Gabra6* expression and its binding by NFI at early, but not later, developmental times [88]. Thus, REST is an early repressor of NFI occupancy, preventing premature onset of NFI binding to the *Gabra6* gene in pre-migratory GCs within the EGL.

Since GC dendrite/synapse formation takes place within the GL additional, post-migratory mechanisms must also be involved in the timing of NFI occupancy. Recent studies identified an NFI developmental “switch” program operating in GCs maturing within the GL [91]. This program consists mainly of several hundred NFI-regulated genes expressed with two distinct time frames: “early” genes downregulated by NFI as the GL matures, and “late,” temporally upregulated genes associated with mature GCs that are activated by NFI [91]. Importantly, many late-expressed genes participate in dendrite/synapse formation and function, directly implicating the NFI switch program as an essential component of GC synaptic maturation.

As observed for *Gabra6*, a central feature of the NFI switch program is delayed NFI occupancy of late genes as GCs mature [91]. So what regulates NFI temporal binding of late genes within the GL? Resting membrane potential is more depolarized in immature GCs and becomes hyperpolarized as the GL matures [92]. Maintaining cerebellar tissue in a depolarizing medium prevents the maturation of GC dendrites in the GL [92]. This depolarization block involves activation of voltage-gated Ca^2+^ channels (VGCCs) and the Ca^2+^-dependent stimulation of calcineurin phosphatase, which inhibits a gene expression switch [93].

**Fig. 4:**
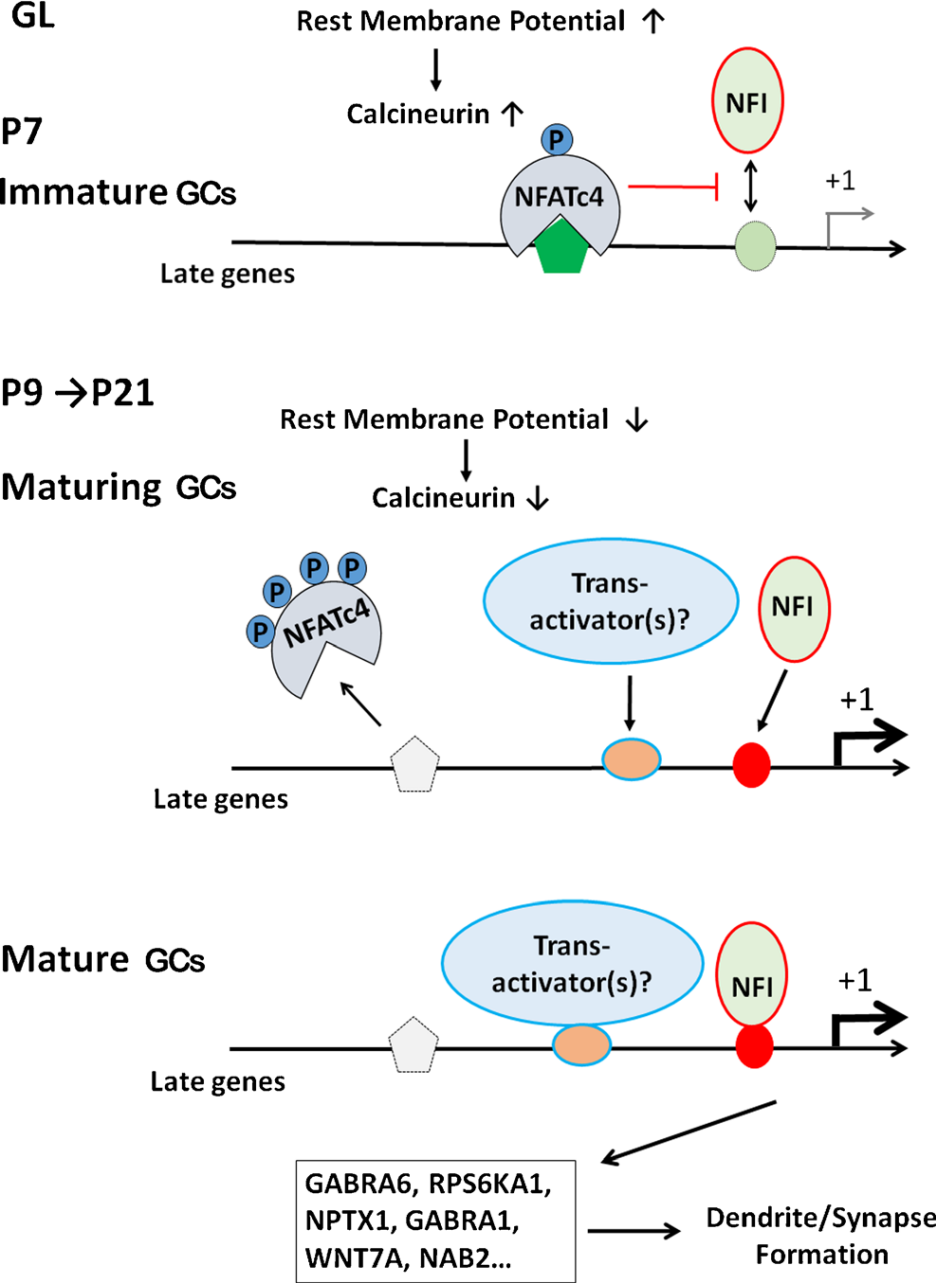
Voltage-sensitive regulation of NFI temporal occupancy in maturing GCs. In the immature GL of the cerebellum (P7), a more depolarized resting membrane potential elevates calcineurin (CaN) activity, leading to NFATc4 nuclear localization and binding to NFI-late genes. NFATc4 occupancy represses late-gene binding and activation by NFI, which is present in the nucleus throughout CGN development. As the cerebellum matures (P9–P21), the resting membrane potential becomes more hyperpolarized and CaN activity and NFATc4 nuclear localization and promoter occupancy decline. This becomes permissive for NFI binding to and activation of late genes in more mature GCs within the GL, promoting dendrite and synapse formation. NFI temporal occupancy of late genes may also be stimulated subsequent to NFATc4 departure by the binding and/or activity of *trans*-activators that also regulate late gene expression. *GL* granular layer, *GCs* granule cells

Voltage-driven mechanisms also regulate the NFI switch [91]. Importantly, depolarization blocks NFI temporal occupancy of late genes via activation of VGCCs and calcineurin [91]. Calcineurin activity is elevated in the immature cerebellum and declines with development, consistent with its inhibitory role during GC dendritogenesis in vivo [91]. Calcineurin promotes NFAT nuclear localization, and NFATc4 mediates the actions of calcineurin on the NFI switch by binding to late genes in immature GCs and repressing NFI occupancy [91]. This led to a model in which declining calcineurin activity and resultant NFATc4 departure from late genes in the maturing GL becomes permissive for the onset of NFI temporal binding and NFI switch programming (Fig. 4). Calcineurin also inhibits GC dendritogenesis via the repressor MEF2A [94]. Thus, transcriptional de-repression plays an important role in initiating GC dendrite formation.

Finally, late-expressed *trans*-activators (e.g., ETV1 [95]) also may control the timing of NFI binding and dendritogenesis in the developing GL, subsequent to NFATc4 departure (Fig. 4). This may provide greater flexibility in the temporal expression and function of distinct gene subsets during GC dendritogenesis-synaptogenesis.

### Unipolar Brush Cells (G. Sekerková)

**Fig. 5:**
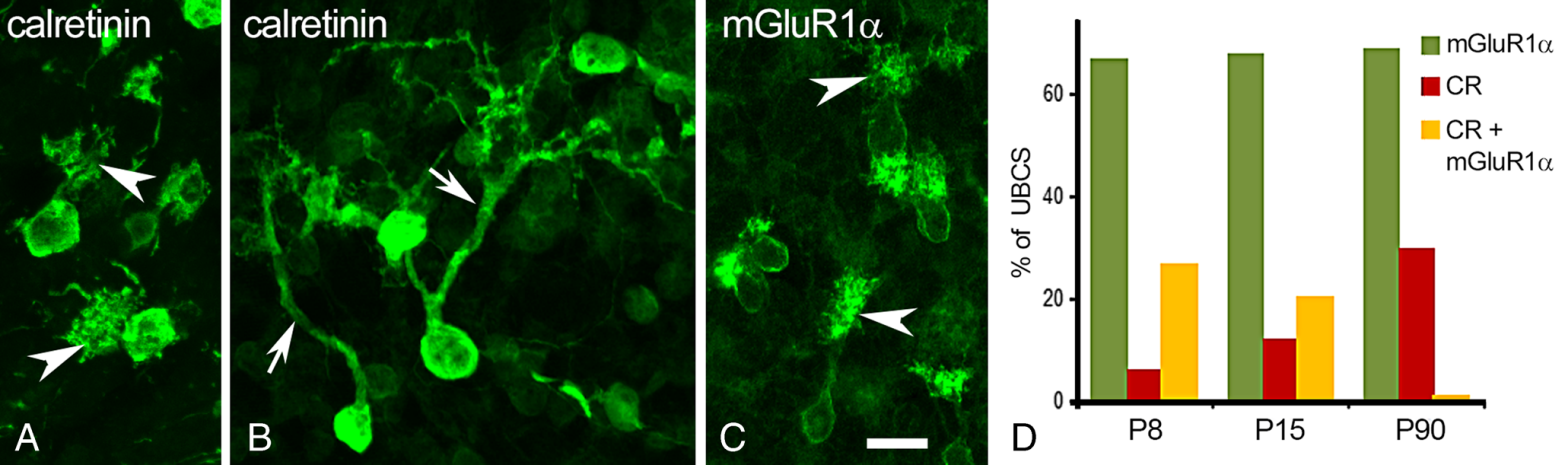
UBCs visualized by cell type specific markers. **a** The typical morphological features of UBCs; a short thick dendritic shaft and brush-like dendrioles (*arrowheads*). Calretinin immunolabeling of a P28 mouse cerebellum. **b**, **c** Images obtained from long-term organotypic mouse cerebellar cultures. In this experiment, the cerebellar nodulus was isolated at P8 and kept in culture for 22 days. During this time, most of the CR-positive type I UBCs (**b**) instead of a dendritic brush have long, branching processes (*arrows*). Under the same condition, all mGluR1α-positive type II UBCs develop distinct brushes (*arrowheads*). *Scale bar* in (**c**) = 10 μm and applies to panels (**a**)–(**c**). **d** Developmental regulation of rat cerebellar UBC chemotype. The UBC fraction expressing only mGluR1α (*green bars*) remains about the same between P8 and P90. However, at P8, a substantial fraction of UBCs expresses both calretinin and mGluR1α (*yellow bars*). These double labeled cells become rare at P90. By P90, UBCs either express calretinin (type I UBCs) or mGluR1α (type II UBCs). The *red bars* represent the UBC fraction expressing only calretinin

UBCs are a class of small excitatory INs associated with the cerebellar cortex and cerebellar-like structures [96–100]. They are especially enriched in the flocculonodular lobules of the cerebellum [100, 101] and the dorsal cochlear nucleus [100, 102, 103], regions that process sensorimotor signals regulating head, body, and eye position. Although our knowledge of UBCs derives mainly from studies in rodents, the main features of UBCs are highly conserved across species: UBCs are already recognized in teleosts and are found virtually unchanged in all mammals, including humans [100, 104–111]. A typical UBC has a single thick dendrite ending in a brush of fine dendrioles, which form a specialized giant synaptic junction with a single mossy fiber terminal (Fig. 5a; [100, 112]). UBC axons branch locally within the GL where they create an intrinsic mossy fiber system superimposed on the canonical extrinsic mossy fiber system [100, 113, 114]. All UBCs are characterized by these morphological features, yet they are classified into type I and type II UBCs, which represent two chemically and functionally distinct subclasses [102, 115, 116] that correspond to the previously identified calretinin (CR)-positive and metabotropic glutamate receptor (mGluR) 1α-positive UBCs, respectively [100, 101, 117–121].

Birthdating studies with bromodeoxyuridine labeling revealed that the two UBC types are also generated within different although overlapping time windows [120]. In rats, type I UBCs are born around embryonic day E16–19 (~E14–17 in mouse) while type II UBCs are produced over a longer period of time extending from E18 (in mouse E16) presumably to early postnatal days (P) 0–1. Initially, they were suggested to originate from a hotspot in the EGL [122], but subsequent histological studies indicated a VZ origin [123]. The definite origin of UBCs was however revealed by genetic fate mapping studies. Englund and colleagues [27] using TBR2, a T-domain transcription factor, as a marker showed that the UBCs—just as for the other glutamatergic neurons of the cerebellum and cochlear nucleus [25, 26]—originate from ATHO1-expressing progenitors of the RL. The UBC progenitors also express WNT1 early in development (E10.5–13.5), but the expression is downregulated before UBCs migrate from the RL [124]. The newly generated UBCs remain in the RL for an additional 1–2 days after which they exit through a narrow channel and migrate to their final destination [27, 38, 125]. The cerebellar UBCs migrate dorsally through the white matter and avoid the cell masses of future CN neurons. Most UBCs reach the GL by P10, several days before GC neurogenesis is complete [27]. The final differentiation of UBCs occurs between P2 and P28. Morin et al. [126] divided the maturation of type I UBCs into four distinct stages based on the morphological appearance of the dendritic brush (the protodendritic, filopodial, intermediate brush, and dendriolar brush stages). In the last stage (P21–P28), the UBCs already exhibit mature UBC morphology and, although their brush keeps expanding, it does so without qualitative changes in the dendriolar pattern. Unpublished data (Sekerková et al.) suggest that somato-dendritic differentiation of the two subtypes is also differentially regulated. In long-term organotypic cerebellar cultures (from P8 mice; 20–25 days in culture), only type II UBCs develop brushes while most of type I UBCs produce long, “branching” dendrites (Fig. 5b, c). Moreover, in vivo, type I UBCs undergo chemical changes (Fig. 5d). Around the first postnatal week, a subset of UBCs co-expresses both CR (the marker of type I) and mGLUR1α (the marker of type II UBCs) and fewer than 10 % of UBCs express only CR. By the third week, the number of double-labeled UBCs decreases dramatically (they virtually disappear by 2 months of age; [100, 115]) and concomitantly the number of the purely CR-positive UBCs increases (to ~33 % of total UBCs). This suggests different postnatal differentiation mechanisms for the UBC subtypes. The differentiation of the brush and the downregulation of mGLUR1α in type I UBCs seem to coincide with the establishment of the first synaptic contacts with external mossy fibers (~P12).

## Patterning of the Cerebellar Cortex: Ventricular Zone-Derived Phenotypes

GABAergic neurons of the cerebellar cortex comprise projection PCs and local INs. All these cells derive from progenitors pools (PCPs and PIPs, respectively) located in different VZ subregions (see “Glutamatergic Phenotypes” section).

### Purkinje Cell Migration (T. Miyata)

Morphologically, PCPs span the ventricular (apical) and pial (basal) surfaces of the cerebellar primordium, taking an elongated (neuroepithelial or radial glial) shape [127]. How PCPs behave is not well understood: whether they divide symmetrically or asymmetrically, how they undergo interkinetic nuclear migration, and the morphology of newly generated PCs in the VZ all remain unknown. Also, whether (and how) a single class of PCPs changes in a temporally regulated manner to generate the different PC subclasses or whether PCPs are inherently heterogeneous within VZ needs to be studied.

Nascent PCs from E14 in mice form a layer several cells thick called the PC plate [128, 129]. This transient structure normally spreads through the presumptive cerebellar cortex until late embryonic days, followed by PC-monolayer formation during the early postnatal stage triggered by RELN signaling (see “Development of Cerebellar Compartmentation” section). The supply of PCs to the cortex occurs sequentially [129, 130]. PCs generated at E10, especially ones born at the posterior VZ close to the RL, are the first to finish their migration and form the plate [131, 132]. This may be because the distance between their birthplace and the goal is the least among the entire PC population (no more than ~200 μm). PCs generated at E11 join the plate from E15, followed by E12-born PCs from E16. These later-born PCs (as well as PCs generated anteriorly at E10) need to migrate over much longer distances than the posteriorly E10-born PCs (up to 700–800 μm in mice).

The early/posterior-born PCs take a tangential migratory route (which is parallel to the pial surface) until E13. They are characterized by long leading processes (100 μm or longer), which morphologically and molecularly resemble axons, and a much shorter trailing process containing the Golgi apparatus. This tangential migration crosses radial glial fibers and is reminiscent of that exhibited by RL-derived cells. The initial departure of this early/posterior-born population from the VZ and the subsequent tangential migration are normal in the cerebellum of *reeler* mice. The early/posterior-born PCs then change orientation by sending the original Golgi-rich trailing process into the cortical region that intensely expresses RELN (produced at E13 by RL-derived tangentially migrating cells of the nuclear transitory zone, NTZ) [131]. This switchback-like, tangential-to-radial orientation change between E13 and E14 is strictly dependent on RELN. These observations suggest a short-range action of RELN. This model is supported by co-culture experiments showing that PCs (both normal and *reeler*-derived) will align in vitro along an artificial RELN-rich zone [133]. However, transgenic *reeler* mice artificially expressing RELN under the control of the *nestin* promoter showed an apparently normal (rescued) arrangement of PCs [134], suggesting that RELN may regulate PC behavior in a context-dependent manner.

In contrast to the posterior-born PCs, PCs born more anteriorly exhibit radially oriented somatal morphologies during migration. The close spatial relationship between these PC somata and radial glial fibers supports the prevailing model that PC migration is guided by radial glial fibers [129, 135]. These radially oriented PCs at E12 or E13 have axon-like fibers that ascend towards the pia [131], while post-migratory (late embryonic) PCs forming the plate have downward-directing axons [129, 130, 136]. Several points remain to be elucidated: how these anterior-born PCs proceed the mid-embryonic migration processes, whether these cells are also affected by dynamic changes of cellular orientation or polarity, and how RELN contributes to histogenesis by these radially oriented PCs.

### Development of Cerebellar Compartmentation (M. Arancillo, R. Hawkes, R. V. Sillitoe)

The fundamental architecture of the cerebellum is an elaborate pattern of transverse zones and parasagittal stripes [137, 138] that is highly reproducible between individuals and conserved across birds [139, 140] and mammals (reviewed in [141, 142]). Compartmentation is revealed by intrinsic differences between subsets of PCs (e.g., zebrin II/aldolase C (ZII [143]; phospholipase C (PLC) β3/4 [144]; HSP25 [145]), the restriction of INs (reviewed in [146]), patterns of pathological PC death (reviewed in [147]), the phenotypes of multiple cerebellar mutants (e.g., *lurcher* (*Grid* ^*Lc*^)—[148]; *rostral cerebellar malformation* (*Unc5c* ^*rcm*^)—[149]; *weaver* (*Kcnj6* ^*wv*^)—[150]; *cerebellar deficient folia* (*Ctnna2* ^*cdf*^)—[151]), and the topography of afferent and efferent projections (reviewed in [152]).

Cerebellar compartmentation appears to start at ~E10 in the VZ of the fourth ventricle but not earlier, e.g., [153–156]. PC subtype specification likely occurs when PCs undergo terminal mitosis between E10 and E13 [54] in the *Ptf1a*-expressing progenitor domain of the VZ ([23, 34, 49]: see “Glutamatergic Phenotypes” section). Birthdating studies have identified two distinct PC populations: an early-born cohort (E10–E11.5) destined to become zebrin II (ZII)^+^ and a late-born cohort (E11.5–E13) destined to become ZII^−^. A direct correlation is also found between PC birthdates and their adult stripe location, suggesting that both subtype specification (e.g., ZII^+^ vs. ZII^−^) and positional information (which zone or stripe) are acquired at this time, e.g., [30, 157–160]; both are cerebellum-intrinsic and not activity- or afferent-dependent, e.g., [161–164]. However, there is no reason to believe that individual PC stripes have a clonal origin. During the same interval, the cerebellar anlage undergoes a 90° rotation, which converts the embryonic rostrocaudal axis into the mediolateral axis of the cerebellar primordium [156]. This suggests the possibility that the adult mediolateral stripe array derives from the anteroposterior patterning of dorsal r1.

A *Ptf1a*-N*eurogenin 1*/*2* (*Neurog1*/2)-*Early B*-*cell factor 2* (*Ebf2*) regulatory network is implicated in PC subtype specification [49]. By this model, the early-born PC cohort expresses neither *Neurog1*/*2* nor *Ebf2* and therefore expresses the ZII^+^ phenotype in the adult. Soon after E11, *Neurog1*/*2* is upregulated by *Ptf1*a in the later-born PC progenitors (e.g., [36]). In this context, neurogenin 2 regulates cell-cycle progression, neuronal output, and early dendritogenesis of PC progenitors [50], but neither *Neurog1* nor *Neurog 2* deletions affect cortical patterning (Hawkes, unpublished observation). In turn, *Neurog1*/*2* ^+^ precursors express EBF2, which represses the ZII^+^ phenotype ([132, 159]: *Ebf2* deletion results in transdifferentiated PCs that express markers characteristic of both the ZII^+^ and ZII^−^ subtypes—the only manipulation known to alter a PC subtype phenotype). In addition, *Ebf2* plays an anti-apoptotic role in ZII^−^ PCs by locally regulating *Igf1* gene expression [165]. As a result of these events, early-born PCs become ZII^+^ in the adult and late-born PCs adopt the ZII^−^ phenotype.

Postmitotic PCs migrate dorsally from the VZ, in part along radial glia processes ([51]: also see Section 4.1), and stack in the cerebellar plate with the earliest-born (Ebf2^−^) PCs located most dorsally. Starting at ~E14, the cerebellar plate undergoes a complex redisposition, such that by E18 a reproducible array of clusters of multiple molecular phenotypes is present on each side of the midline ([166–170], etc.: reviewed in [171, 172]) . The cellular processes that guide cluster formation are not understood but grafts of dissociated PCs also organize into discrete ZII^+/−^ compartments [173], pointing to cell-cell adhesion molecules as possible organizers: cadherins are strong candidates reviewed in [174]. Over 50 distinct clusters have been identified [175, 176]. The mapping between embryonic clusters and adult stripes is complex: in some cases, one cluster yields a single stripe, e.g., [175, 177, 178], but in others single stripes derive from the fusion of several clusters, e.g., [179], or single clusters split into multiple stripes, e.g., [136].

**Fig. 6:**
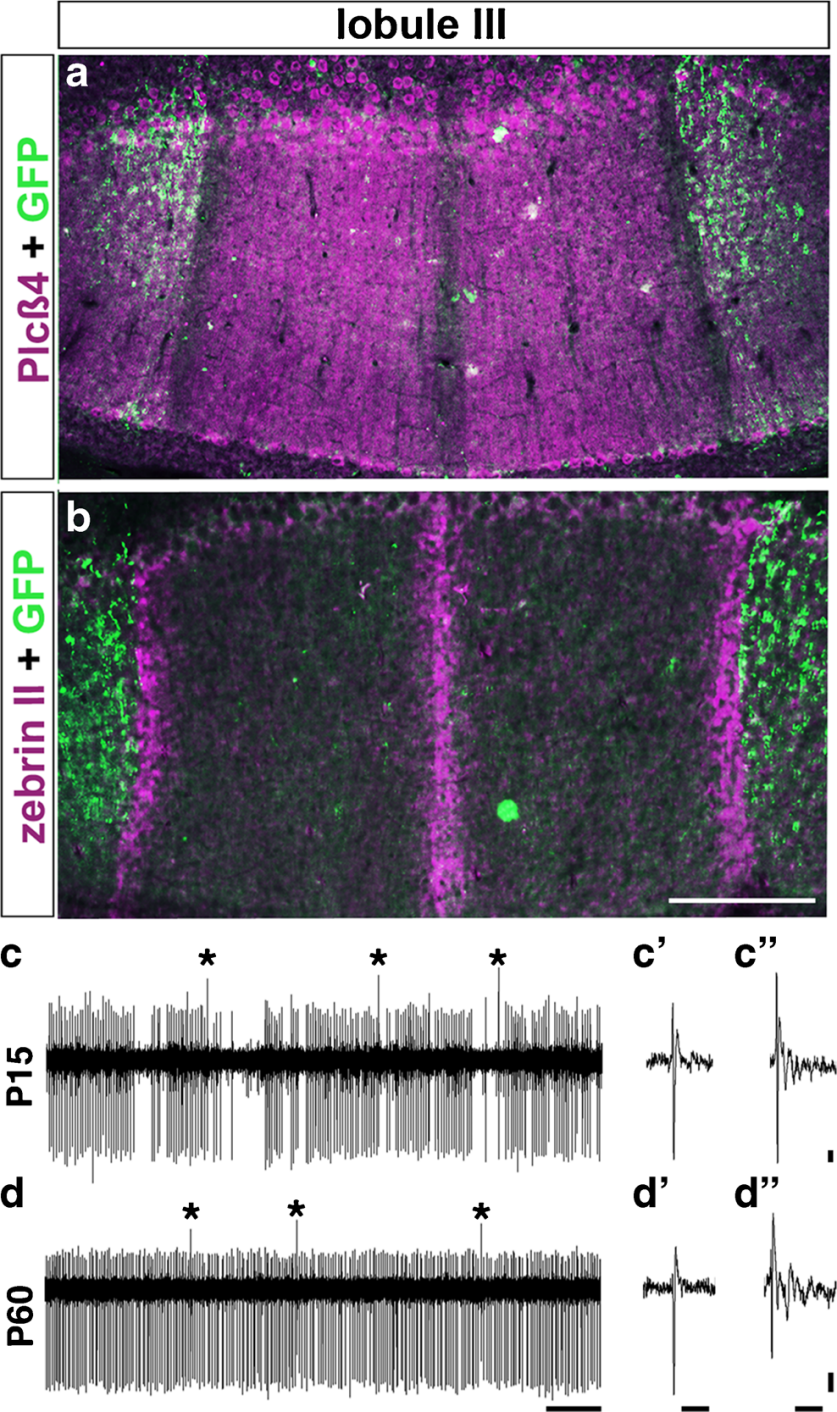
Purkinje cell organization and firing activity can define how cerebellar circuit topography is assembled. **a** CF zones, which were labeled with the transgenic marker *Npy*-*GFP* (*green*), are arranged in broadly spaced parasagittal stripes from the midline and overlap with PC zones that are immunopositive for the marker PLCβ4 (*magenta*). **b** *Npy*-*GFP*-labeled CF zones (*green*) are restricted according to PC stripe boundaries and do not cross zebrinII-immunopositive stripes (*magenta*). *Scale bar* = 250 μm. **c** At P15 in mouse, PCs fire simple spikes with a relatively low firing rate and an irregular “burst” pattern (as shown in sample trace). **c**′ An example of a simple spike at P15. **c**ʺ An example of a complex spike at P15. **d** PC activity is dynamically sculpted during key events of circuit formation, until a mature firing pattern (as shown in sample recording trace at P60) is established at about 4 weeks of age. **d**′ An example of a simple spike at P60. **d**′’ An example of a complex spike at P60. *Scale bar* in d: x = 0.5 s. *Scale bars* in **c**′, **c**′′, **d**′, and **d**′′: x = 5 ms, y = 2 mV. (**a**) and (**b**) were adapted from [191]. (**c**) and (**d**) were adapted from [214]

The embryonic cluster architecture is the scaffold around which other cerebellar elements are organized. First, clusters likely restrict the distribution of cerebellar INs and their processes (GCs—[180]; Golgi cells—[181]; stellate/basket cells—[146]; UBCs—[182]: reviewed in [146]: also glia—e.g., [183, 184]). Secondly, most afferent projections also enter the cerebellum between E14 and E18 and target specific PC clusters (the so-called matching hypothesis—reviewed in [185]). There are two major sources of sensory input to the cerebellum: CFs from the inferior olive, and mossy fibers from a number of brain and spinal cord regions. Both afferent systems invade the cerebellum at around E 13/14 in mouse [186, 187], and thereafter they terminate with a spatial organization that parallels the pattern of PC stripes [137, 188] (Fig. 6a, b). PC subtype organization is thought to play a key role in instructing circuit wiring into topographic maps. Spontaneous and engineered mouse mutants that display disrupted PC stripes have equivalent alterations in the spatial arrangement of mossy fiber and CF terminals [189–191]. But, what molecular mechanisms trigger cluster dispersal and wiring?

From ~E18, the embryonic PC clusters disperse, triggered by RELN secreted by the EGL [192–194]: recently thoroughly reviewed in [195]. RELN is critical as its deletion in the *reeler* mouse (*Reln* ^*rl*^: [192, 196] blocks dispersal, e.g., [128, 197]). One model is that secreted RELN binds two surface receptors on PCs—Apolipoprotein E receptor 2 and the Very Low Density Lipoprotein Receptor. Deletion of either receptor (*Lrp8* ^*tm2Her*^ or *Vldlr* ^*tm1Her*^) produces a partial, stripe-specific disruption of cluster dispersal [198]: deletion of both receptors blocks dispersal [199]. In turn, RELN binding induces receptor clustering [200] and activates two Src-family kinases—Fyn and Src [201, 202]—which tyrosine phosphorylate [203, 204] the intracellular adaptor docking protein Disabled1 (DAB1—[205–209]: phosphorylation is essential and key tyrosine point mutations phenocopy *reeler*—[210]). DAB1-phosphorylation results, at least in the neocortex, in Repressor activator protein 1 (Rap1)-mediated homophilic cadherin 2 cell-cell interactions that promote neuronal migration [211, 212]. The upshot is that by ~P20, as cerebellar lobulation matures, the PC clusters string along the rostrocaudal axis into the adult array of long parasagittal stripes. However, one critical question is how do these dynamically changing stripes acquire their functional properties during development?

During postnatal development, chemo- and activity-dependent mechanisms may play important (and possibly distinct) roles in establishing the afferent topographical map. There is a long-standing hypothesis that first a “crude” topographic map is established by genetic cues. And one of the most compelling hypotheses postulates that cues in the afferent source domains match up with cues in the PC map [185]. The molecular cues that would mediate this mechanism have not been resolved, although it may involve a chemoaffinity mechanism mediated by eph/ephrin and cadherin signaling. But, one has to also consider how the circuit is then sculpted into a “fine” map, and does the mechanism for refining the map also involve PC patterning? Alternatively, are there non-genetic mechanisms that also contribute? Indeed, it was recently shown that the striped patterning of PCs is disrupted when neurotransmission in the PCs themselves is selectively silenced [213]. Interestingly, the patterning of spinocerebellar mossy fiber terminals into distinct stripes was also altered in the absence of PC activity. The sharp stripe boundaries that are typically observed were severely compromised, although the basic features of the topographic map were left intact. These data argue that neuronal activity may play an important role in fine-tuning the cerebellar map into topographic domains. But maybe PCs are not the only players. Perhaps inhibitory INs in the developing molecular layer (ML) or the millions of excitatory GCs also influence circuit topography by modulating the levels of PC activity. INs, after all, are also organized into stripes [146] and they integrate into the PC microcircuit at a time when they could have a powerful affect on how the circuit is firing when it is wiring [31].

Which features of neuronal firing need to be examined in order to understand how circuits assemble? PCs provide the best starting point since these cells exhibit two distinct types of action potential that are experimentally tractable with in vitro and in vivo paradigms. The first are complex spikes that are triggered by CF inputs, and the second are simple spikes generated intrinsically within the PCs but modulated by mossy fiber inputs (Fig. 6c–cʺ, d–dʺ). Recent work in anesthetized and awake mice used in vivo electrophysiology to record PC activity in postnatal mice [214]. The study found that the rate of complex spike firing increased sharply at 3 weeks of age, whereas the rate of simple spike firing gradually increased until 4 weeks of age. They also found that compared to adult, the pattern of simple spike firing during development was more irregular as the cells tended to fire in “bursts” that were interrupted by long pauses (Fig. 6c, d). The regularity in simple spike firing only reached maturity at 4 weeks of age. These data show that PC activity is dynamically sculpted throughout postnatal development, traversing several critical events that are required for circuit formation. Importantly, the establishment of PC firing properties seems to overlap with the final stages of stripe maturation [139]. However, there are also data that support an alternate view which suggests that activity-dependent mechanisms may not be involved in topographic wiring. Surgically lesioning the neonatal spinocerebellar afferent tracts did not induce a competitive sprouting of the adjacent cuneocerebellar pathway [215] and the regression of supernumerary CFs appears spatially and temporally unlinked to the formation of stripe patterns—both processes may be strictly dependent on molecular cues [216]. Therefore, it is interesting to speculate that compared to molecular cues, the rate and pattern of spikes, at particular ages, could shape a more subtle level of topography by refining the connectivity within stripes rather than between individual stripes. If this was to be the case, then chemical tags might define the fundamental patterns of stripes irrespective of sensory experience, and perhaps only later during postnatal development is when activity might tune and complete the existing map.

### GABAergic Interneurons (K. Leto, E. Parmigiani, K. Schilling, A. Wefers)

**Fig. 7:**
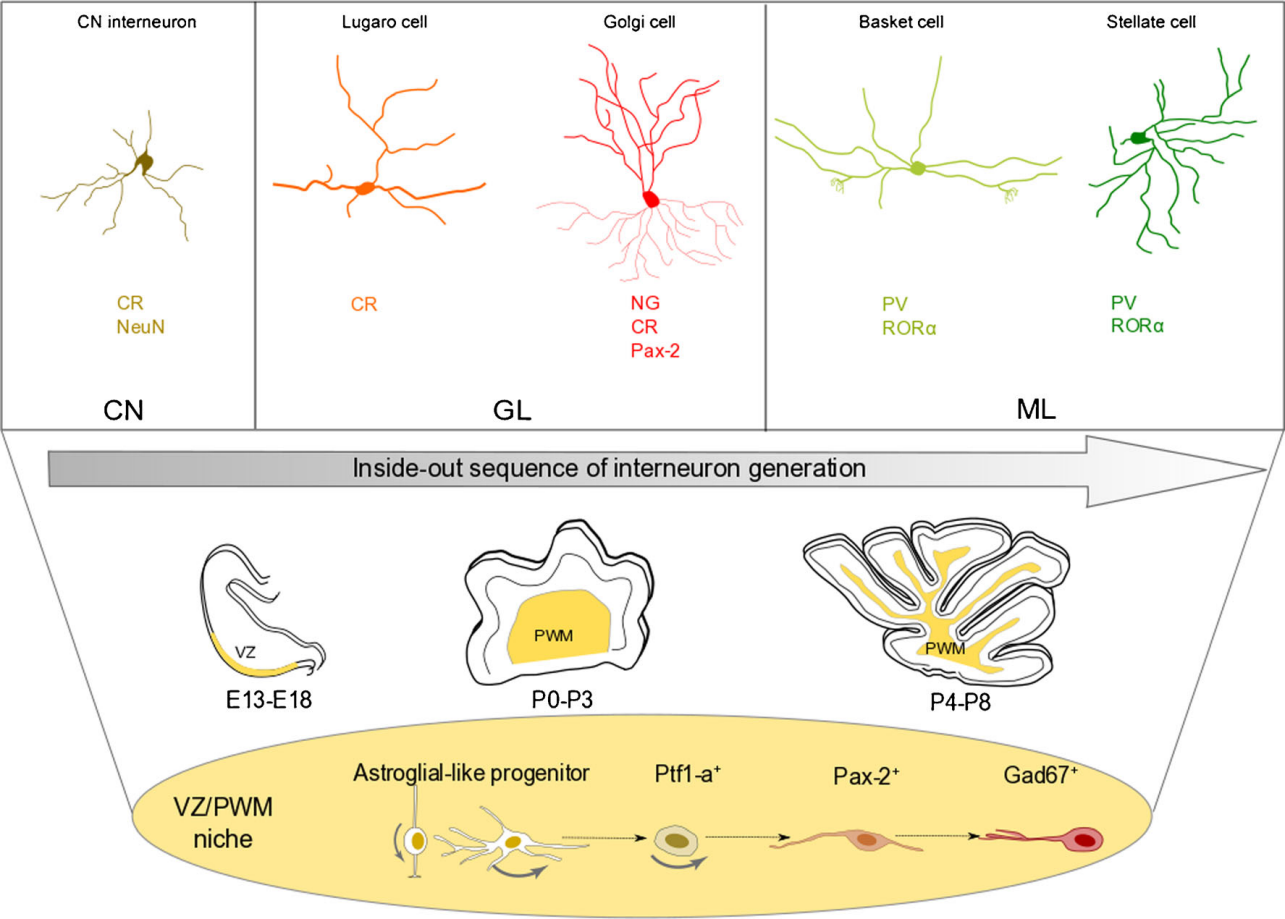
Production of inhibitory interneurons. Different subtypes of GABAergic INs are present at different levels of the cerebellar cortex and CN and are characterized by the expression of specific markers. All these cells are produced through an inside-out sequence from common Pax-2^+^ progenitors residing in the VZ/PWM niches during cerebellar development. Interneuron progenitors derive from astroglial-like bipotent progenitors, which also give rise to parenchymal astrocytes (see text). *CN* deep cerebellar nuclei, *GL* granular layer, *ML* molecular layer, *VZ* ventricular zone, *PWM* prospective white matter

The term “GABAergic interneuron” is traditionally used to refer to a diverse set of neurons that, in the healthy adult cerebellum, result in local inhibition (Fig. 7). Actually, it is somewhat of a misnomer since in CN and the GL, the majority of GABAergic INs also use glycine as a co-transmitter, and a few are strictly glycinergic [217, 218]. Distinct differences in their afferent and efferent wiring, their morphology, and the differential expression of a set of molecular markers (inter alia mGluR2 and neurogranin) allow us to distinguish, in the GL, at least four discrete sets of Golgi cells sensu stricto as well as Lugaro and globular cells. While these clearly distinct inhibitory INs of the GL are sometimes collectively referred to as Golgi cells, INs of the ML are traditionally classified as basket or stellate cells. Today, the weight of the evidence rather supports the view that these two terms describe two exemplary variants of one population that shows gradual morphological [219] and molecular [220] differences which may be secondary to their position in the ML (discussed in [146]). Lastly, the still rather enigmatic candelabrum cell [221] may be yet another member of the basket/stellate class (see [222] for a broader discussion).

GABAergic INs are produced from late embryonic life to the second postnatal week, according to a precise inside-out sequence (Fig. 7). The proliferation of inhibitory INs peaks during the first postnatal week and exclusively occurs in the PWM, a postnatal niche containing heterogeneous cell types at different maturation stages [29, 32, 223–227]. CN INs are the first to be born during embryonic and early postnatal life, followed by GL INs (Golgi and Lugaro cells) and, finally, by those of the ML (basket and stellate cells; [29, 31, 225, 228]).

The first studies of cerebellar neurogenesis postulated that ML INs derive from the EGL, the only germinal layer known at that time to be active during postnatal development [229, 230]. Later, analysis of chick-quail chimeras, transplantation experiments, and retroviral injections demonstrated that the EGL exclusively generates GCs and indicated that all the GABAergic neurons, including the ML INs, derive from the VZ [154, 231–234]. While these diverse cells all originate from the VZ, there is little evidence that this germinal layer is pre-patterned, say by differential gene expression, such as to presage the diversity of cerebellar inhibitory INs in the adult. One possible exception may be the expression of Neurog1, which may distinguish the lineages leading to cerebellar cortical and CN inhibitory INs ([235, 236]: the interpretation of these results is ambiguous because the BAC used may not faithfully recapitulate cognate *Ngn1* expression, specifically in CN [235]). In contrast, there is compelling experimental evidence that PIPs maintain a high degree of plasticity and acquire their definitive fate only as they migrate through the deep cerebella mass (i.e., the PWM) on their way into the CN or the nascent cerebellar cortex [237]. This is particularly striking as individual subsets of cerebellar cortical inhibitory INs withdraw from the cell cycle over an extended period from E13 through the second postnatal week. However, cells collected at any point through this long generative phase are capable, when heterochronously transplanted, of acquiring a fate temporally appropriate to the host tissue [237]. Significantly, fate determination by transplanted cells is critically dependent on migration through the recipient’s PWM. These findings have led to the recognition of the nascent white matter as an instructive niche critical for the maturation and diversification of cerebellar inhibitory INs [237]. Unfortunately, we are still quite ignorant as to how this instruction is realized on the molecular level. There is experimental evidence that PC-derived SHH regulates proliferation of precursors of cerebellar inhibitory INs in and near the ventricular epithelium [33, 238]; yet, whether PCs also influence the diversification of these cells remains unclear.

The dividing intermediate progenitors that are responsible for the extensive amplification of the inhibitory interneuron populations strongly express Ptf1a [33]. At later stages, the precursors of inhibitory INs are seen as a population of Pax-2^+^ cells that appears in the VZ around E12.5 and later moves into the cerebellar parenchyma [32, 228].

The precise lineage relationships linking Ptf1a^+^ interneuron progenitors to the other precursor pools in the PWM (namely, progenitors of astrocytes and oligodendrocytes) remained obscure for many years. The existence of multipotent progenitors in the postnatal cerebellum has been proposed by two independent studies [239, 240], in which the isolated progenitors were able to form neurospheres and to differentiate into neurons, astrocytes, and oligodendrocytes both in vitro and in vivo after grafting into newborn mice [239, 240].

A series of more recent studies have clarified the properties of PWM progenitors and the relationships between the different lineages. For instance, it has been shown that the postnatal cerebella of mice lacking the proneural gene *Ascl*-*1* have fewer PAX-2^+^ INs and increased numbers of SOX9^+^ astrocytes compared to controls [241]. Conversely, overexpression of ASCl-1 in the VZ results in more INs at the expense of astrocytes [241], suggesting the existence of lineage relationships between these cell types throughout cerebellar development. It is not clear whether similar relationships between INs and astrocytes are also present at earlier embryonic ages. Indeed, previous fate mapping analyses tagging embryonic progenitors producing PCs or INs only rarely generated astrocytes [34, 50], suggesting that the bulk of cerebellar astrocytes do not derive from these progenitors. Subsequent lineage analysis during embryonic and postnatal development showed that both INs and astrocytes derive from ASCL-1^+^ precursors [31]. Similarly, fate-mapping studies of progenitors with astroglial traits—such as the expression of hGFAP [242], TenascinC [33], and GLAST [227]—reveal that the progeny of such progenitors often comprise a mixed population of astrocytes and GABAergic INs. In particular, in the study of Fleming et al. [33], a population of primary CD133^+^TenC^+^ astroglial progenitors was identified in the PWM as the putative source of both PTF1-a^+^ intermediate IN progenitors and CD15^+^ intermediate astrocyte precursors. The existence of these bipotent progenitors has been confirmed by a recent study in which mixed clones of INs and astrocytes were derived from PWM astroglial progenitors both in vitro and in vivo [227].

Finally, PCs are critical for the terminal differentiation and morphogenesis of cerebellar INs. Specifically, the complexity of basket/stellate cell axonal arborizations and their positioning on PCs is critically dependent on neurofascin [243, 244] and also Semaphorin (Sema3a)/neuropilin-1-mediated signaling between PCs and differentiating ML INs [245]. Further, the preferential orientation of basket/stellate cell axons in the sagittal plane may be due to PC guidance, as they extend in the ML [146]. Conversely, dendritic differentiation of basket/stellate cells appears primarily sensitive to GC-derived input, including BDNF [246] and signaling through the GluD1 receptor. In fact, ablation of this receptor, which is highly expressed in ML INs and concentrated at their synapses with PFs, results in reduced survival and stunted growth of early post-migratory ML INs [247].

### Development of the Cerebellar Nuclei (R. J. T. Wingate)

The description of cerebellar development above is focused on the cerebellar cortex. The CN develop in parallel, using much of the same molecular machinery. The CN dictate the participation of the cerebellum in a range of circuits by providing an almost exclusive efferent connectivity via axon pathways to more caudal structures (from medial nuclei) and more rostral structures projections (from lateral nuclei; [248]). Their largely spontaneously active output can be excitatory or inhibitory and nuclei contain several different local interneuron types [249–253]. Nuclei receive collateral input from afferents to the cerebellar cortex (CFs and mossy fibers) in addition to inhibitory input from PCs. Decoding both the development and integrative function of nuclear circuits is likely to be critical for understanding the broader function of the cerebellum. Despite their functional significance, our knowledge of CN development is incomplete. Furthermore, the number of CN varies between tetrapod species suggesting that the developmental mechanisms responsible for their patterning are a key locus of evolutionary adaptation [254].

The perhaps surprising absence of a detailed description of CN specification and maturation can to some extent be explained by a major conceptual revision of their development over the last 10 years following insights from a series of studies of gene expression [51, 60] and genetic fate-maps [25, 26, 34, 255]. Prior to this, CN, which condense initially in a “nuclear transitory zone” at the margin of the cerebellar anlage, were assessed by birthdate to be derived exclusively from the VZ [256]. This assumption was overturned by genetic lineage maps using an *Atoh1* reporter showing that excitatory CN neurons arise by tangential migration from the RL prior to GCP production [25, 26]. More recent mapping using a *Ptf1a* reporter [36] reveals that the VZ gives rise only to inhibitory neurons of the CN. Hence, just as for the cerebellar cortex, the assembly of CN is defined as the coordinated integration of PTF1a^+^ and ATHO1^+^ lineages in local circuits. However, patterns of temporal specification in either lineage suggest important differences compared to the cerebellar cortex in how these lineages interact.

**Fig. 8:**
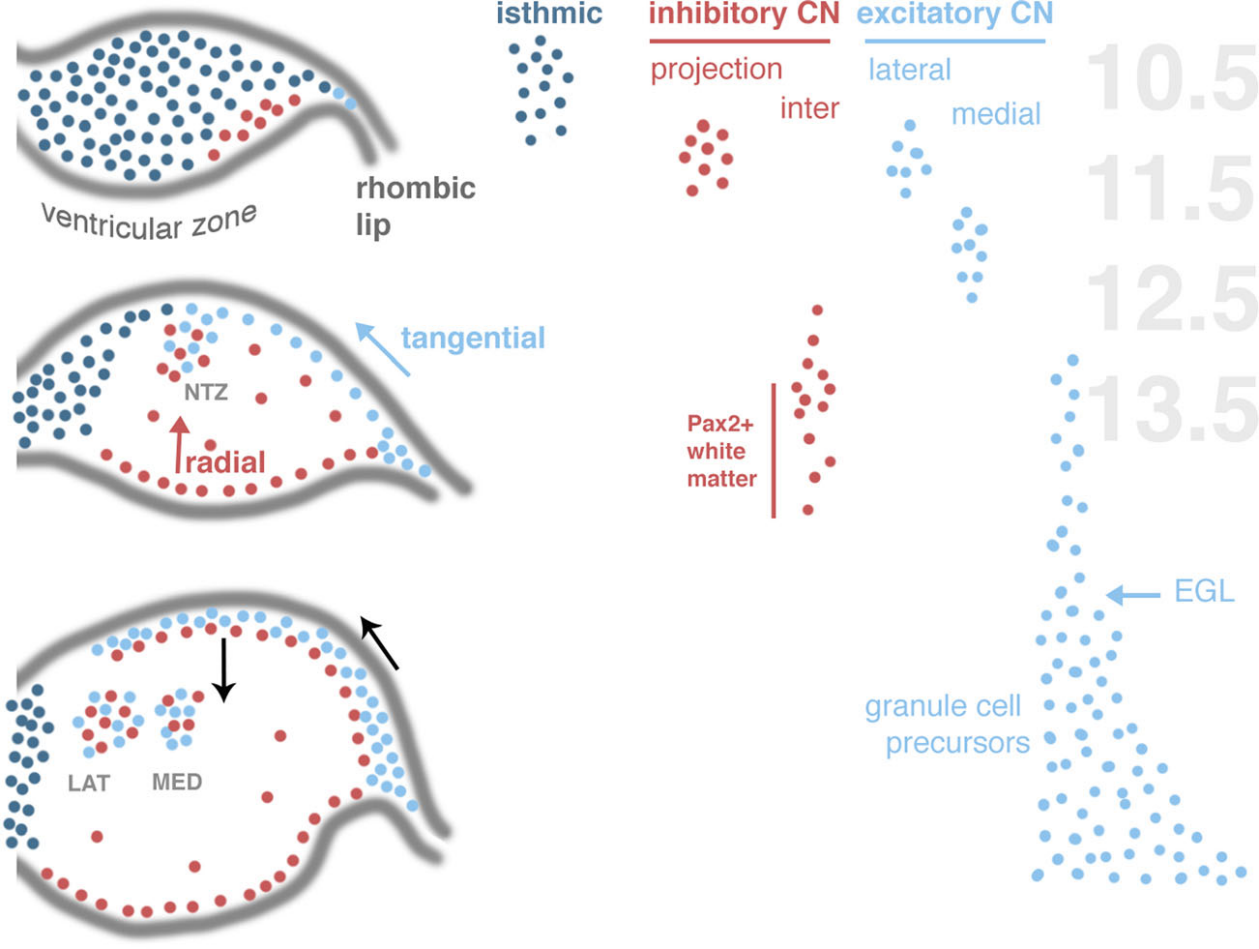
The assembly of cerebellar nuclei requires the complex spatiotemporal integration of neurons from Atoh1^+^ and Ptf1a^+^ lineages. Schematic profiles to the left plot the distribution of the Atoh1^+^ lineage (FGF-dependent, *dark blue*; BMP-dependent, *light blue*) and Ptf1a^+^ lineage (*red*) in the developing embryo (after [456]). To the right, the approximate timeline of different neuron groups is shown next to embryonic days in mouse. *Light-blue* and *red arrows* indicate tangential and radial migration of, respectively, excitatory and inhibitory CN neurons. *Black arrows* show the direction of the CGPs. *NTZ* nuclear transitory zone, *CN* deep cerebellar nuclei, *EGL* external granular layer, *lat* lateral, *med* medial

*Ptf1a*-derived CN neurons are generated as the first of a sequence of inhibitory neurons destined for progressively more superficial fates [29]. To some extent, this reflects a progressive dorsal expansion of a Gsx1^+^/Ptf1a domain that gives rise to INs, which occupy the *Olig2*/*Ptf1a* domain and give rise to PCs (see “Specification of Cerebellar Progenitors” section). However, fate determination in the former *Pax*-*2* ^+^ lineage is notably a product of local microenvironmental factors. Migrating PIPs persist in the white matter, continuing to contribute to the CN ([29, 36]: see “GABAergic Interneurons” section) well beyond the production of the first, inhibitory projection neurons (Fig. 8). By contrast, cell fate in the ATHO1^+^ CN derivatives of the RL not only correlates with birthdate [25, 26, 257] but appears determined at the RL [258]. This raises the possibility that the temporal pattern of RL derivatives establishes a template around which GABAergic neurons are organized.

The allocation of a temporal framework of RL-derived CN components is accompanied by a characterized sequence of transcriptional maturation [51, 60] that results in a first born LHX9^+^ lateral nucleus (projecting to midbrain and thalamus), followed by a TBR1^+^ medial (fastigial) group, which sends axons to the hindbrain via the fasciculus uncinatus, or hook bundle [257]. The progressive deposition of cells in more dorsal (ultimately medial) positions reflects a decreasing sensitivity to netrin signaling from ventral midline in migrating cells [259, 260]. Netrin receptors are also responsible for determining the laterality of the projections of CN axons [261], which extend seamlessly from the leading processes of migrating cells [260]. Target selection itself (rostral or caudal CNS) appears to be a property of LHX9 [257] versus TBR1 [59] expression.

If this process of RL cell fate specification provides a template for nucleus assembly, it places a special emphasis on poorly understood events at the NTZ. It is here that neurons segregate into a series of mediolaterally distributed nuclei as they are subducted under the rapidly expanded cortex by either passive displacement, or possibly active translocation [256]. To understand whether lineage interactions are part of this process, let alone underlying mechanisms of nucleogenesis, will rely on a better description of neurons subtypes. For example, it is unclear whether only more lateral nuclei contain inhibitory projection neurons [252, 262] or indeed how much of the repertoire of neurons seen in the lateral nucleus is recruited to other nuclei [263]. Similarly, the embedded nature of nuclei within a series of re-entrant loops that include the inferior olive, in addition to pontine neurons and PCs, has implications for later developmental events. How does the convergence of afferents onto nuclei influence the organization of INs into precise, geometric functional units [249]? These elements of fine-grained developmental detail, which are so significant for the function of the cerebellar circuit, are almost completely unexplored.

### Gliogenesis in the Cerebellum (A. Buffo)

In contrast to other CNS areas in which gliogenesis follows neurogenesis, in the cerebellum the generation of glia parallels the generation of GCs and INs. What triggers the activation of gliogenesis and regulates its course in this territory is still poorly understood.

**Fig. 9:**
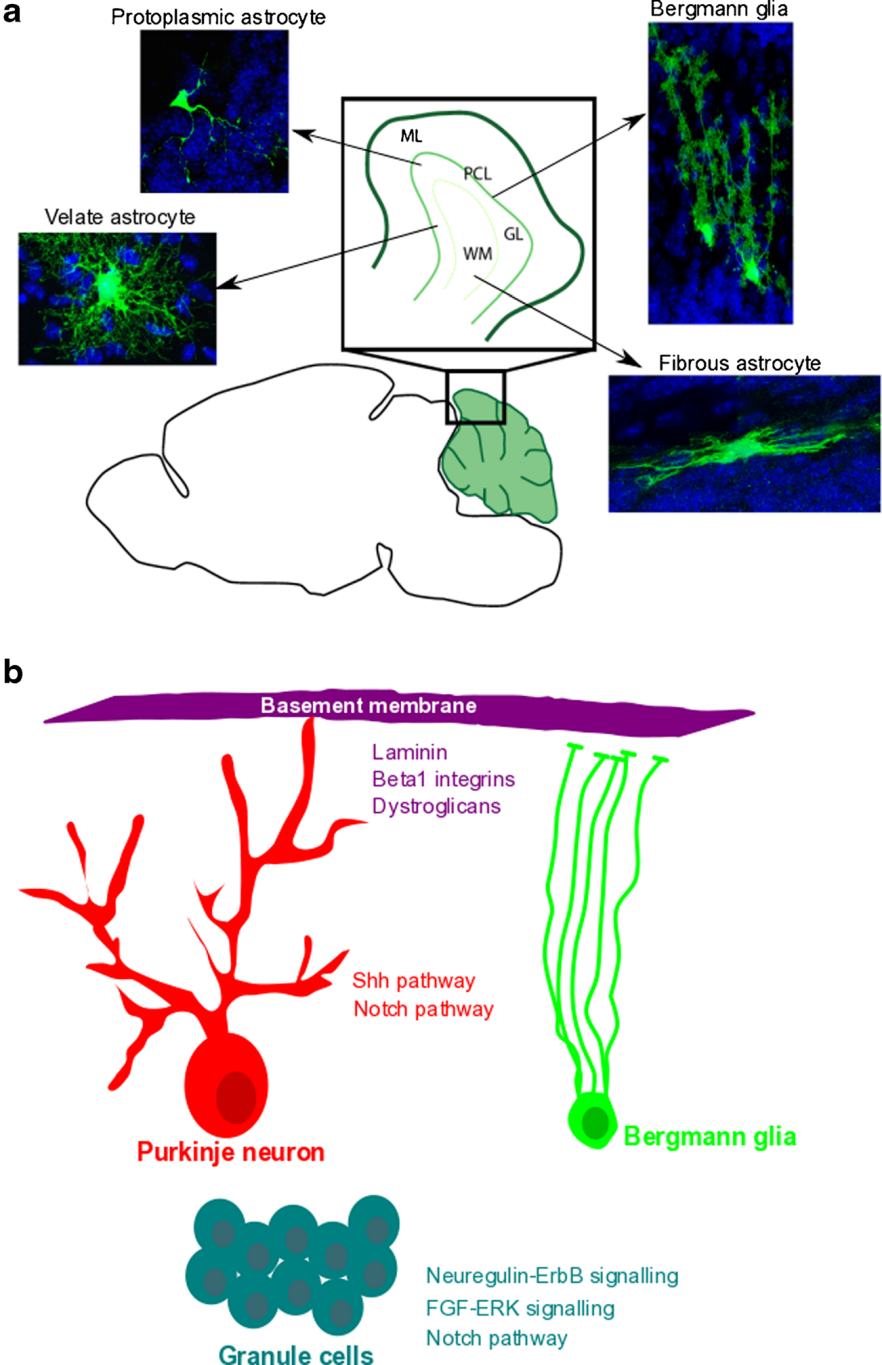
Astrocyte morphological heterogeneity in the mouse cerebellum and factors promoting Bergmann glia maturation. **a** Distinct morphologies define astroglial cell subtypes in the adult mouse cerebellum. **b** Components of the cerebellar microenvironment [270, 457–459] regulate signaling pathways that modulate the acquisition and maintenance of the BG phenotype. *ML* molecular layer, *PCL* Purkinje cell layer, *GL* granular layer, *WM* white matter

In the mature mammalian cerebellum, four astroglial subtypes are classically distinguished, including fibrous astrocytes in the white matter (WM), stellate multipolar astrocytes with profuse tiny processes (velate astrocytes) or more slender morphologies (protoplasmic astrocytes) in the GL, and neuroepithelial cells displaying radial BG basal processes spanning from the cells bodies in the PC layer through the entire ML, up to the subpial basement membrane (Fig. 9a, [130, 229, 264]). In the future, this classification may expand to comprise more astroglial subtypes based on neurochemical, topographical, and morphological criteria, as shown by a recent detailed investigation on the human cerebellum [265].

Through the comparative analysis of different mammalian species, Ramón y Cajal [229] proposed that cerebellar astroglia derive from the VZ. Cajal’s interpretation has been fully proved by means of fate mapping analyses in mice in which radial glial progenitors (RG) at the ventricle were targeted based on the expression of stem-cell markers [266–268], or VZ-restricted tags [34]. A small contribution of the RL to cerebellar astrogliogenesis has also been proposed [269] but so far remains controversial (see [270] and references therein). As showed by anatomical investigations and functional manipulation of regulatory pathways, in a first astrogliogenic wave (up to about E14 in mouse), RG detach from the ventricle and displace the cell body towards the nascent PWM, transforming into progenitors of BG ([229, 266, 271, 272]: the relationship between astrocytes and GABAergic INs is reviewed in “GABAergic Interneurons” section). A subset of these precursors appears to be already postmitotic at the moment of translocation [31] and readily differentiates into BG. Conversely, other precursors form a proliferative layer that expands [130, 266, 271] up to the first postnatal week in parallel with the tangential expansion of the cerebellar surface. At later stages of embryonic development, a second wave of astroglial-like progenitors lacking the basal process delaminates from the VN into the cerebellar PWM where they proliferate, forming astrocytes populating the prospective GL and white matter [130, 271]. Whether PWM astroglial progenitors also produce some BG or whether proliferating BG contribute astrocytes to other cortical layers remains to be established. Similarly, the dynamics and timing of the amplification of astroglial progenitors are largely unknown.

How the specialization of the astroglial subsets is achieved is only partially clarified. BG morphogenesis requires a tight and timely regulated interaction with the surrounding cerebellar microenvironment (basement membrane, PCs, migratory and proliferative GCs, see Fig. 9b). Notably, impairment of these regulatory mechanisms results in BG malpositioning and/or the acquisition of a stellate morphology, which may thus represent a default differentiation pathway for cerebellar astroglial precursors. Yet, it is likely that the refinement of the variety of multipolar morphologies in the GL and WM is instructed by local cues. Further, few intrinsic determinants are known that take part in the establishment of distinct astroglial cerebellar phenotypes (see [270]).

In contrast to astrocytes, so far no evidence clearly demonstrates the derivation of oligodendrocytes from the cerebellar VZ [241]. Rather, a minor oligodendroglial fraction appears to derive from progenitors in subventricular positions, likely residing in the PWM [31, 273]. Alternatively, mouse transplantation experiments indicate an extracerebellar origin for the majority of oligodendrocytes [241]. In line with these data, experiments in chick-quail chimeras and in ovo transplants in the chick brain demonstrated that cerebellar oligodendroglia are generated in the chick mesencephalic neuroepithelium and only subsequently invade the cerebellum via the velum medullare [274]. A similar extracerebellar source in the mammalian brain remains to be identified. Further, fate-mapping analyses [242] support the hypothesis that the majority of cerebellar oligodendrocytes have no lineage relationships with cerebellar astrocytes (and neurons). However, ex vivo experiments [275] and functional deletion of the polycomb group protein Bmi1 [273] pointed to the existence of bipotent gliogenic precursors, whose identity remains to be established.

Once settled in the cerebellar primordium, oligodendrocyte progenitors first surround the CN and gradually invade the nascent cortical lobules, progressing in a centrifugal direction. The same pattern is reflected in the course of both differentiation and myelination that proceed from the inner cerebellar portions to the lobule apices [276–279].

Despite the fact that most intrinsic and extrinsic mechanisms regulating oligodendrocyte differentiation appear to be common to multiple CNS sites, including the cerebellum [280, 281], a particular role in the regulation of the maturation of cerebellar oligodendroglia is exerted by thyroid hormones (TH; L-triiodothyronine, T3; L-tetraiodothyronine, thyroxine, T4; see “The Role of Thyroid Hormone in Cerebellar Development” section) and PC-derived signals. In particular, PCs secrete SHH, which stimulates oligodendrocyte progenitor proliferation at early postnatal stages, whereas by the end of the first postnatal week, they start producing vitronectin, which drives oligodendrocyte maturation and myelin formation [282].

### Extrinsic Regulators of Cerebellar Development: The Role of SHH (C. Chiang)

**Fig. 10:**
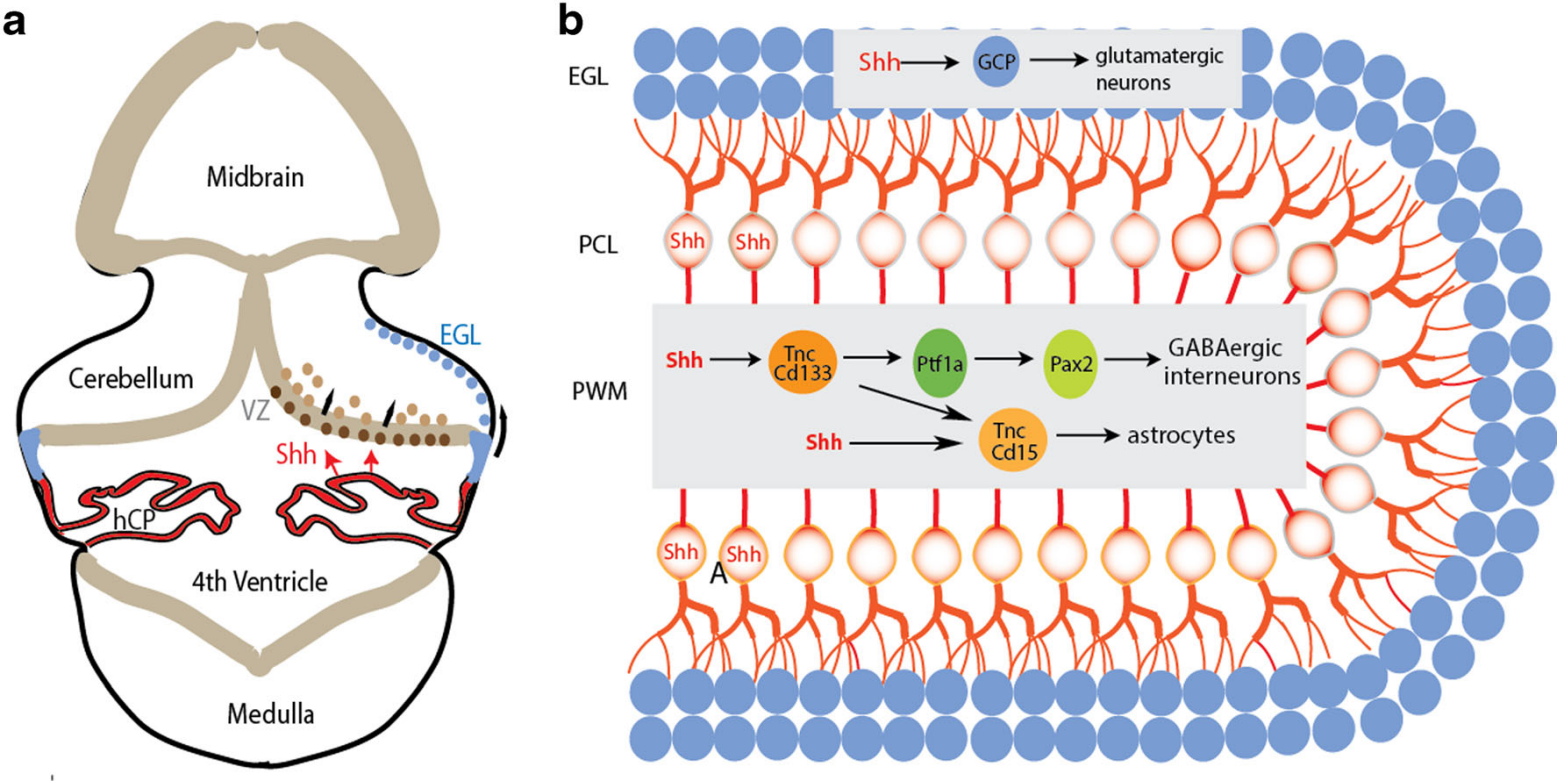
Shh regulates the expansion of functionally diverse neuronal and glial cell types from spatially restricted precursors in the cerebellum. **a** Schematic illustration of a coronal section of the midbrain-cerebellum-medulla region at E14.5. *Beige* regions represent the VZ neuroepithelium. The hChP epithelium, depicted in *red*, secretes Shh protein into the fourth ventricle which is then delivered to the cerebellar VZ to promote the proliferation of its resident radial glial cells (*brown*). The nascent EGL is shown in *blue* (modified from [45]). **b** PC-secreted Shh simultaneously stimulates proliferation of GCPs and stem-like astroglia (Tnc^+^, CD133^+^) located respectively at the distant EGL and PWM. The PWM niche is comprised of lineage-related, but molecularly and functionally divergent progenitor subpopulations that descend from common astroglia (modified from [33]). *VZ* ventricular zone, *EGL* external granular layer, *PCL* Purkinje cell layer, *PWM* prospective white matter, *GCP* granule cell progenitor

The SHH pathway has been extensively studied in the context of GCP proliferation. However, more recent studies have revealed additional roles for this important pathway during different phases of cerebellar development. The common theme emerging from these studies is that SHH is a key mitogen for the expansion of functionally diverse neuronal and glial cell types from spatially and temporally restricted precursors. However, the mechanisms by which SHH stimulates proliferation of these precursors appear to be distinct, involving both cerebellar and extracerebellar strategies (Fig. 10).

In the cerebellum, *Shh* expression is restricted to PCs starting at E16.5 and continuing throughout adulthood [42, 283]. The early phase of *Shh* expression is critical for rapid clonal expansion of GCPs as blockade of *Shh* expression in PCs leads to drastic reduction of GCP number in the EGL [62, 283–285]. The expansion of GCPs requires the cell surface proteins BOC and GAS1 [286], which synergistically promote SHH binding to its receptors PTCH1/2. In the absence of *Shh*, PTCH1 functions as a negative regulator of SHH signaling by suppressing the activity and localization of a seven-pass transmembrane protein, smoothened (SMO) to the primary cilium, a slim, microtubule-based non-motile structure that projects from the surface of nearly all vertebrate cells [287, 288]. Therefore, SHH binding to PTCH1 relieves SMO from inhibition, triggering SHH signaling and subsequently activating downstream target gene expression mediated by the GLI family of transcription factors [289]. Accordingly, mutations in ciliary components that disrupt SMO localization to the tip of the cilium all lead to altered SHH signaling and reduced GCP proliferation [290, 291]. Among the three GLI proteins, GLI2 acts as the primary transcriptional activator in GCPs [292]. In addition to canonical GLI target genes *Gli1*, *Hhip*, and *Ptch1*, several others including *MycN* and *CcndD1* involved in cell-cycle regulation of GCPs have been reported [293, 294]. Notably, cerebellar phenotype in the absence of *MycN* function resembles that of *Shh* mutants [63]. Recent studies show that activation of GLI-dependent target genes is facilitated by JMJD3, a H3k27me3 demethylase involved in epigenetic conversion of inactive to active chromatin state [295], highlighting the importance of chromatin modification in enabling GCPs to respond to SHH during cerebellar development.

While *Shh* expression persists in PCs, GCPs eventually exit the cell cycle and differentiate to GCs. This process, as shown by recent studies, appears to be promoted by the transcriptional repressor BCL6 through recruitment of BCOR co-repressor and SIRT1 deacetylase to the *Gli1* and *Gli2* promoter regions, thus epigenetically silencing their expressions [296]. Indeed, loss of BCL6 impedes the differentiation of GCPs, which however do not continue to proliferate, likely due to p53-mediated cell death. Removal of p53 rescues GCPs from cell death and restores their proliferation. However, it is unclear how BCL6 is activated in the immature GCs [296].

In addition to GCPs, SHH signaling is also required for the expansion of GABAergic INs by regulating precursors in two different neurogenic niches [33, 42]. In the VZ niche, SHH signaling is activated in multipotent radial glial cells after E12.5. Defective SHH signaling in VZ severely impairs the proliferation of radial glial cells and their ability to generate GABAergic interneuron progenitors during the embryonic period [42]. Conversely, persistent activation of SHH signaling greatly expands their numbers. The source of SHH signal acting on VZ radial glial cells appears to be extracerebellar as SHH expression is not yet established in the emerging PC population. Indeed, SHH is prominently expressed in the hindbrain choroid plexus epithelium (hCPe), a secretory organ whose development is in close apposition with the cerebellar VZ (Fig. 10a). The presence of SHH protein in the circulating embryonic cerebrospinal fluid suggests that SHH is actively secreted from the hCPe and delivered to the adjacent VZ via a transventricular mechanism. Further support for this model comes from the observation that VZ progenitor proliferation is compromised in mice with reduced *Shh* expression in the hCPe [42].

The cerebellar VZ is also the source for stem-like astroglial cells of the secondary neurogenic niche residing in the PWM during late embryonic and postnatal period [33, 239, 242]. These astroglial cells transiently respond to PC-derived SHH and express cell surface marker CD133 (also referred to as Prominin) as well as extracellular matrix glycoprotein Tenascin-C (Tnc) [33]. Lineage analysis reveals that the PWM astroglial cells generate intermediate progenitors of both astrocytes and GABAergic INs as marked by the expression of CD15 and PTF1a, respectively (Fig. 10b) [33]. Furthermore, attenuation of SHH signaling in astroglia during the neonatal period leads to a significant reduction of both intermediate progenitor classes, underscoring the importance of SHH signaling in the maintenance of the PWM niche. Surprisingly, PTF1a-expressing GABAergic progenitors represent an additional population that responds to SHH in PWM [33, 238]. In contrast to cerebellar VZ [42], these PTF1a-expressing progenitors are proliferative and likely responsible for rapid expansion of the late-born GABAergic INs during the first week of the postnatal period [225].

### Cerebellar Foliation (A. L. Joyner)

**Fig. 11:**
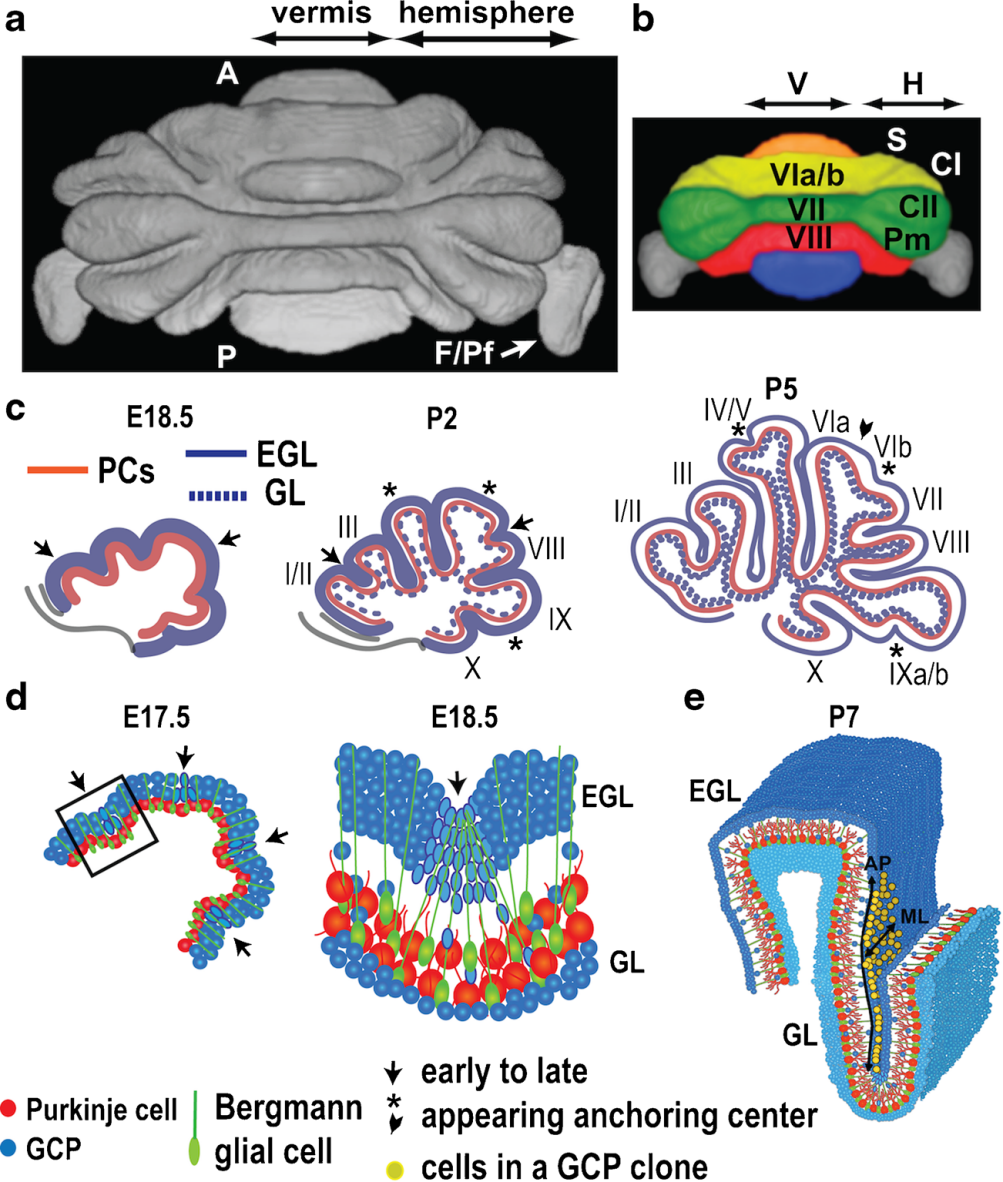
Cerebellar foliation. **a** 3D rendering of MRI image of mouse cerebellum segmented at the EGL surface. *A* anterior, *P* posterior, *F/Pf* flocculus/paraflocculus. **b** Lobules that are continuous between the medial vermis (V) and lateral hemispheres (H) are color-coded. (**a**) and (**b**) provided by Kamila Szulc and Daniel Turnbull. **c** New anchoring centers and associated fissures form on particular days as the folia grow outward (modified from [64]). **d** Changes occur in the PC, GCP shape, and BG fiber projections in anchoring centers. Sagittal sections through the vermis (**c**, **d**), with lobules indicated in *roman numerals* [300]. *S* simplex lobule, *CI/II* Crus I and II of the ansiform lobule, *Pm* paramedian lobule. **e** Clonally derived GCPs (*yellow*) do not cross the bases of fissures and disperse more along the AP axis than medial-lateral (ML) axis and have a greater cell number in long lobules compared to short lobules (provided by Emilie Legué). *EGL* external granular layer, *GL* granular layer, *GCP* granule cell progenitor

The most striking morphological feature of the cerebellum of birds, mammals, and some fish is its foliation pattern, or subdivision into lobes, lobules, and sublobules that are separated by a series of fissures [130, 171, 297, 298] (Fig. 11a). In most species, the foliation pattern is symmetrical with respect to the midline, and the fissures run perpendicular to the anterior-posterior (AP) axis in the medial cerebellum (vermis) of most species. The mammalian cerebellum is further subdivided into two lateral hemispheres and adjacent flocculi/paraflocculi, each with distinct foliation patterns with fissures that vary in their orientation. Nevertheless, the lobules of the hemispheres are continuous with lobules in the vermis (Fig. 11b). Larsell proposed a unified scheme for naming the lobules in the vermis of birds and mammals with roman numerals I–X from anterior to posterior [299, 300]. To account for the variation in foliation pattern between species and the fact that the cerebellum has more than ten lobules in many species, lobules were subdivided into sublobules separated by shallower fissures (e.g., VIa and VIb, Fig. 11c). The vermis of mice has eight or nine lobules, as lobules I/II are not separated in some strains and IV/V are fused, and the hemispheres have four lobules [301, 302] that extend laterally from lobules VI and VII (Fig. 11b, c). The basic pattern of vermis foliation is conserved throughout mammals, but foliation in the hemispheres is more variable than in the vermis and is very complex in primates (see discussion in Chapter 1 of [130], [303]). As the cerebellum modulates the functions of all areas of the neocortex [304], by extrapolation the development of the two brain regions should have co-evolved. Indeed, the entire spinocerebellar tract projects only to the medial cerebellum, thus the hemispheres are enriched for connections to the neocortex. Furthermore, although the volume of the cerebellum as a percentage of the total brain is constant across species [305, 306], the greatest proportional increase in brain regions has occurred in the cerebellum and neocortex [306], and the ratio of the number of neurons in the cerebellum to the neocortex is remarkably constant across mammalian species [307]. It is therefore tempting to speculate that during evolution particular lobules in the hemispheres and folds (gyri) in the neocortex of gyrencephalic mammals that house interconnected neural circuits have arisen and expanded in unison [308]. Defining the circuits between the cerebellum and neocortex in primates as well as rodents is a major challenge and high priority for future cerebellar research, but dependent on development of effective tools for tracing across multiple synapses. It will be exciting to trace the axon pathways from a parasagittal stripe of PCs in one region of a particular lobule through to the neocortex and back to the cerebellum. One hypothesis is that this will reveal an elaborate spatial organization of neurons, with ones dedicated to similar functions being housed in distinct lobules in the cerebellum and gyri in the neocortex, and with new circuits discovered specific to humans.

The cerebellum undergoes its major growth in the third trimester and infant stage in humans, and the first 2 weeks after birth in mice, primarily due to expansion of GCPs [130, 309, 310]. The surface area of the cerebellum increases during development much more than its volume due to the formation of lobules [311–314]. The lobules thus serve to house a large number of neurons in a layered cytoarchitecture in a small area. Foliation begins at E16.5 in the mouse with the sequential formation of the base of each fissure, which we have termed anchoring centers [315]. The GCPs, PCs, and BG within anchoring centers have distinct characteristics, and the lobules grow out away from them [314, 315]. The first sign of formation of an anchoring center is an inward thickening of the EGL that is followed by formation of an indentation of the outer surface of the cerebellum and elongation of the bodies of the GCPs (Fig. 11d, [315]). The underlying PC layer indents and then the surrounding fibers of BG project to the base of the fissure. In the mouse vermis, four initial anchoring centers form that defines five initial lobes, which are further subdivided. Based on mutant analysis, the timing of formation and position of two adjacent anchoring centers define the morphology of the intervening lobule and thus the allocation of cells available for distinct long-range circuits [315–317]. Importantly, the homeobox engrailed genes (*En1*/*2*) are fundamental to the patterning process as they determine when particular anchoring centers form [315–320]. A number of theories have been proposed for how foliation is regulated [130, 315, 321–323]. Fundamental questions remain, such as whether one cell type initiates formation of anchoring centers and how are they positioned.

Clonal analysis previously uncovered that GCPs divide symmetrically to expand each clone and then differentiate en masse [324]. Surprisingly, from a clonal analysis of GCPs, we recently found that the anchoring centers act as lineage restrictions that prevent GCPs from moving between lobules [325]. One possibility is that the restriction in GCP movement produces a mechanical force driving the lobules outward. Furthermore, the number of cells per clone in long lobules is almost twice that in short ones. In terms of clone geometry, the length is greater in the AP than medial-lateral axis in all EGL clones, especially in long lobules, accounting for the tremendous AP expansion of the cerebellum (Fig. 11e). Moreover, in *En1*/*2* mutants with smaller lobules, the size and geometry of clones is similar to wild-type clones in short lobules [325]. Thus, the dynamics of GCP expansion is differentially regulated in lobules with different shapes/sizes. Whether this is a cell intrinsic property of GCPs that form different lobules that is established before anchoring centers are formed remains to be determined. What forces within and outside the cerebellum impact on the foliation process is an additional critical question to address. A further question is how scaling of all cell types in the cerebellum is regulated to ensure that the correct proportions of all neurons/glia are allocated to each lobule. One interesting possibility is that SHH secreted by PCs [285, 292] determines the expansion of INs and astrocyte/BG progenitors in unison with regulating GCP proliferation [33, 64, 285, 326].

The cerebellum arose in gnathostomes, but the emergence of a stable transient amplifying population of GCPs in an EGL structure that is stimulated by SHH seems to date to the transition to amniotes [326–332]. While the production of the enormous number of granule neurons (>50 % of all neurons in mouse and human [333, 334]) is thought to drive foliation [130] and has been experimentally linked to foliation, e.g., [64, 335], it is important to point out that some sharks have extensively foliated cerebella [336]. Interestingly, the degree of foliation seems to correlate better with the ecological environment and/or complexity of prey behavior within and between clades rather than with phylogeny [306, 336]. An important question to resolve is whether the ratio of GCs to PCs is increased in sharks with highly foliated cerebella. Although it is not known whether SHH stimulates neurogenesis and of what cell types in sharks, it will be interesting to determine whether the SHH pathway has been co-opted to increase GC production in sharks since *Shh* has been detected in PCs of a shark but not in zebrafish [327]. Furthermore, given the different cytoarchitecture of the shark cerebellum, the mechanisms underlying foliation could be distinct from that in amniotes. Determining the similarities and differences in the foliation processes between sharks and amniotes and their implications for circuit allocation will be valuable, and likely to have implications for formation of folds in the neocortex of gyrencephalic mammals.

### Refinement of the Climbing Fiber Afferents (M. Kano, N. Uesaka)

PCs in the adult cerebellum receive two distinctive excitatory synaptic inputs—from PFs, the axons of GCs, and from CFs arising from the inferior olivary nuclei in the medulla oblongata. Each PC receives functionally weak but numerous (~100,000 in mice) PF synapses on spines of its distal dendrites. In contrast, most PCs are innervated by a single but functionally very strong CF on the stubby spines of their proximal dendrites. However, in the neonatal cerebellum, each PC is innervated on the soma by multiple CFs [337]. How is the adult one to one relationship between a CF and a PC established during postnatal development?

**Fig. 12:**
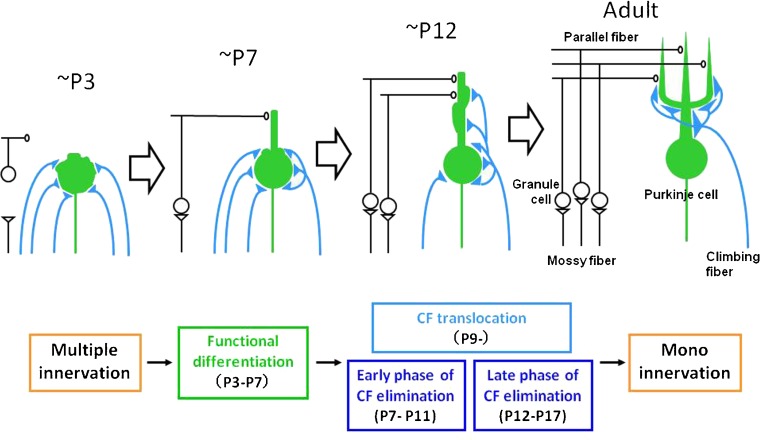
Diagram showing postnatal development of CF-PC synapse. Until P3, synaptic strengths of multiply-innervating CFs are relatively uniform. From P3 to P7, one CF is selectively strengthened, which is termed the phase of “functional differentiation.” From P9 on, the strongest (“winner”) CF undergoes translocation to growing dendrites (the phase of CF translocation). On the other hand, weaker (“loser”) CFs remain around the soma and are eventually eliminated in two distinct phases (the “early and late phases of CF elimination”; from [340]). *CF* climbing fiber

Immature olivocerebellar axons extensively ramify in the white matter and the GC layer, and give rise to many collaterals around PCs (creeper stage) [338]. Since immature PCs have no large primary dendrites, CFs terminate on perisomatic protrusions and thorns emerging from the PC somata. By P2–P3, several individual CFs form multiple synapses with relatively similar synaptic strengths on a single PC (Fig. 12). During the first postnatal week, a single CF is selectively strengthened on the soma of each PC (termed “functional differentiation”). Mice deficient in Cav2.1, the α-subunit of the P/Q-type voltage-dependent Ca^2+^ channel (VDCC), show impairment in the selective strengthening of a single CF, suggesting that activity-dependent Ca^2+^ influx through VDCCs is crucial for establishing a single “winner” CF on each PC [339, 340]. Next, the strongest CF extends its innervation territory from the soma to the dendrites (“CF translocation”: Fig. 12). As mentioned above, CFs initially establish synaptic contacts on the fine processes emerging from the soma and form a plexus (“pericellular nest” stage) [229]. As the dendrites of the PCs start to grow into the ML, from around P6, multiple CFs continue to innervate the PC somata until P9. After the functional differentiation of CFs, only the “winner” CF extends its innervation territory from the soma to the stem dendrites from P9 (“capuchon” stage) [229]. In the “dendritic” stage [229], CF synapses undergo progressive translocation to the growing PC dendrites. In contrast, the “loser” CFs remain around the soma and are eventually eliminated in two distinct phases (the “early and late phases of CF elimination”) mediated by distinct mechanisms [216, 339, 340]. The early phase of CF synapse elimination starts at around P7 soon after the functional differentiation is completed. Unlike the late phase of CF synapse elimination, the early phase is not dependent on the proper generation of GCs and PF-PC synapses. Several lines of evidence suggest that neuronal activity is crucial for this event [339, 340].

The late phase of CF synapse elimination starts at around P12 [216, 339, 340]. This process is critically dependent on the proper formation of excitatory PF synapses and inhibitory basket cell synapses on PCs. In mice deficient in mGluR1 or any of its downstream signaling molecules (Gαq, PLCβ4, PKCγ), the late phase of CF elimination is severely impaired. The immediate early gene *Arc*/*Arg3.1*, the neurotrophin receptor TrkB, and insulin-like growth factor 1 are also involved in CF synapse elimination [339, 340]. A recent study has revealed that postsynaptic Sema7A, a GPI linked subtype of Semaphorin, and its receptors (ItgB1 and PlxnC1) on CFs are involved in the cascade downstream of mGluR1 [341]. In contrast, Sema3A, a secreted class of Semaphorin, and its receptors (PlxnA4) on CFs maintain both weak and strong CFs from P8 to P18, and therefore oppose synapse elimination [341]. Thus, semaphorins mediate retrograde signals from PCs to CFs that regulate multiple processes of CF synapse elimination.

### Dendritic Differentiation of Purkinje Cells (I. Dusart)

The PC stands as a neuronal model to study dendritic differentiation. In addition to being beautiful, PCs are also popular because of their convenience in terms of ease of immunohistochemical detection and genetic manipulation. Proteins such as calbindin or IP3R (inositol phosphate 3 receptor) are specifically abundantly expressed in PCs and label their dendritic tree, soma, and axon. Grafting experiments have provided major progress in our understanding of the biology of neurons [342]. The L7/pcp2 promoter drives gene expression specifically in PCs and retinal rod bipolar neurons [343]. Combining the L7 promoter and an inducible CRE/loxP system with in utero electroporation allows the specific regulation of gene expression in PCs in a temporally controlled manner [344]. Furthermore, different viral vectors target PCs specifically [345] for review. Last but not least, for the study of the development of the dendritic tree, PCs can develop in organotypic cultures. By using this technique, the morphology of individual PCs can be studied since they are isolated from their neighbors [346, 347].

**Fig. 13:**
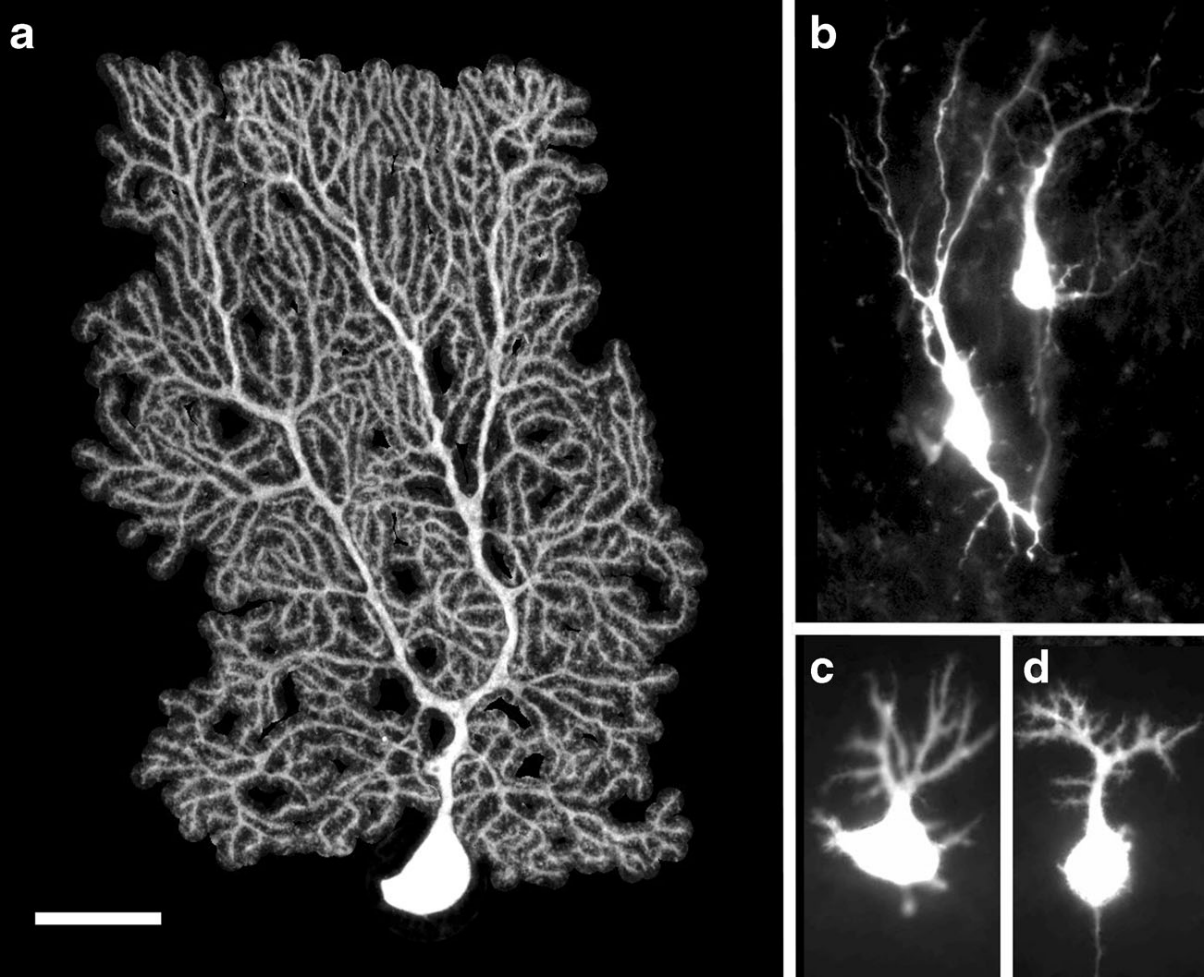
Dendritic differentiation of Purkinje cells. PCs were filled with biocytin using patch-clamp pipette and revealed by incubation with streptavidin coupled to fluorochrome cy3 from 2-month-old (**a**), newborn (**b**), 5-day-old (**c**), and 7-day-old (**d**) mice. (**a**) was imaged with a confocal microscope and its morphology reconstructed with *Neuronstudio* software. The tree model obtained was used as a mask to extracted single PCs from the background image. (**b**, **c**) and (**d**) were imaged with an epifluorescence microscope. *Bar*, 30 μm

Adult PCs are highly recognizable by their large dendritic tree with prototypical morphological characteristics (Fig. 13a). One characteristic of the PC dendritic tree is that its extension and ramification occurs in the sagittal plane, resembling an espaliered fruit tree [229]. This highly stereotyped and simple architecture in a two-dimensional plane is likely at the origin of the PC’s popularity: many neurobiologists, among them Ferdinando Rossi, have been fascinated by their beauty, e.g., [348]. The development of this spectacular dendritic tree occurs during the first three postnatal weeks of the mouse life. Interestingly, it is not a linear process as there is a clear discontinuity at the end of the first postnatal week. During the first postnatal week, successions of growth and retraction have been described [349, 350]. Immature PCs present a panel of very different morphological forms (Fig. 13b,c). In the absence of time-lapse analyses, the relations between these different morphological forms are not yet well understood. It is only from the second postnatal week on that PCs develop their characteristic dendritic trees (Fig. 13d). At the beginning of the second postnatal week, the PCs have a single stem segment at the apical pole that already presages the form of the mature dendritic tree. During the second postnatal week, and up to the end of the third postnatal week, the dendritic tree grows first wider and then taller [351]. By using virus-mediated gene transfer followed by three-dimensional reconstruction of confocal images of labeled PCs, Kaneko et al. [352] demonstrated that PCs achieve their monoplanar configuration by the dynamic remodeling of an initially irregular arrangement extended in multiple sagittal planes during the third postnatal week in mice.

The transition between these two morphological developmental phases—a first phase of intense remodeling and a second phase of continuous development of the mature dendritic tree—occurs in parallel with profound functional transitions [353, 354]. As they occur in parallel with a circulating peak of TH and the acquisition of the ability to walk outside the mother nest, we have proposed that these transitions are reminiscent of amphibian metamorphosis [354, 355].

Numerous intrinsic or environmental factors regulate the dendritic development of PCs (for reviews see [342, 345, 356, 357]) and indeed, understanding the development and the maintenance of the dendritic tree is far from being accomplished. Recent studies of PCs have shed light on new mechanisms. For example, the PC is one of the rare types of neurons in which factors involved in the maintenance of dendritic tree have been identified [358]. In mammals, mechanisms of dendritic self-avoidance, a critical process in patterning neural circuits during development, has also been reported in [359, 360]. Last, in parallel to the classic neurotrophic theory for axons, developing PC dendrites compete for limiting amounts of Neurotrophin-3 (NT3) and require anterograde NT3 from their presynaptic partners in order to grow [361].

### Neurodevelopmental Disorders of the Cerebellum

#### Developmental Malformations (W.B. Dobyns, P. Haldipur, K. J. Millen)

Numerous cerebellar malformations have been described in humans, primarily classified by MRI studies, and can occur in isolation or as part of a broader malformation syndrome involving multiple systems. Most cause cognitive in addition to motor and sensory integration deficits [362, 363]. Cerebellar developmental mechanisms are well conserved between humans and rodents, making studies in mice highly informative towards defining pathogenic mechanisms. Notably, however, cerebellar development in humans begins around the ninth gestational week and continues beyond birth. This protracted developmental timeline makes the human cerebellum particularly vulnerable to insult, especially during 24–40 weeks of gestation, when massive neurogenesis in the EGL causes a fivefold increase in size of the cerebellum [364]. Thus, while several malformations have a genetic basis, inflammation, fetal hemorrhage, and prematurity are often contributing factors. Here, we discuss some of the common and best understood human cerebellar malformations and their causes.

**Fig. 14:**
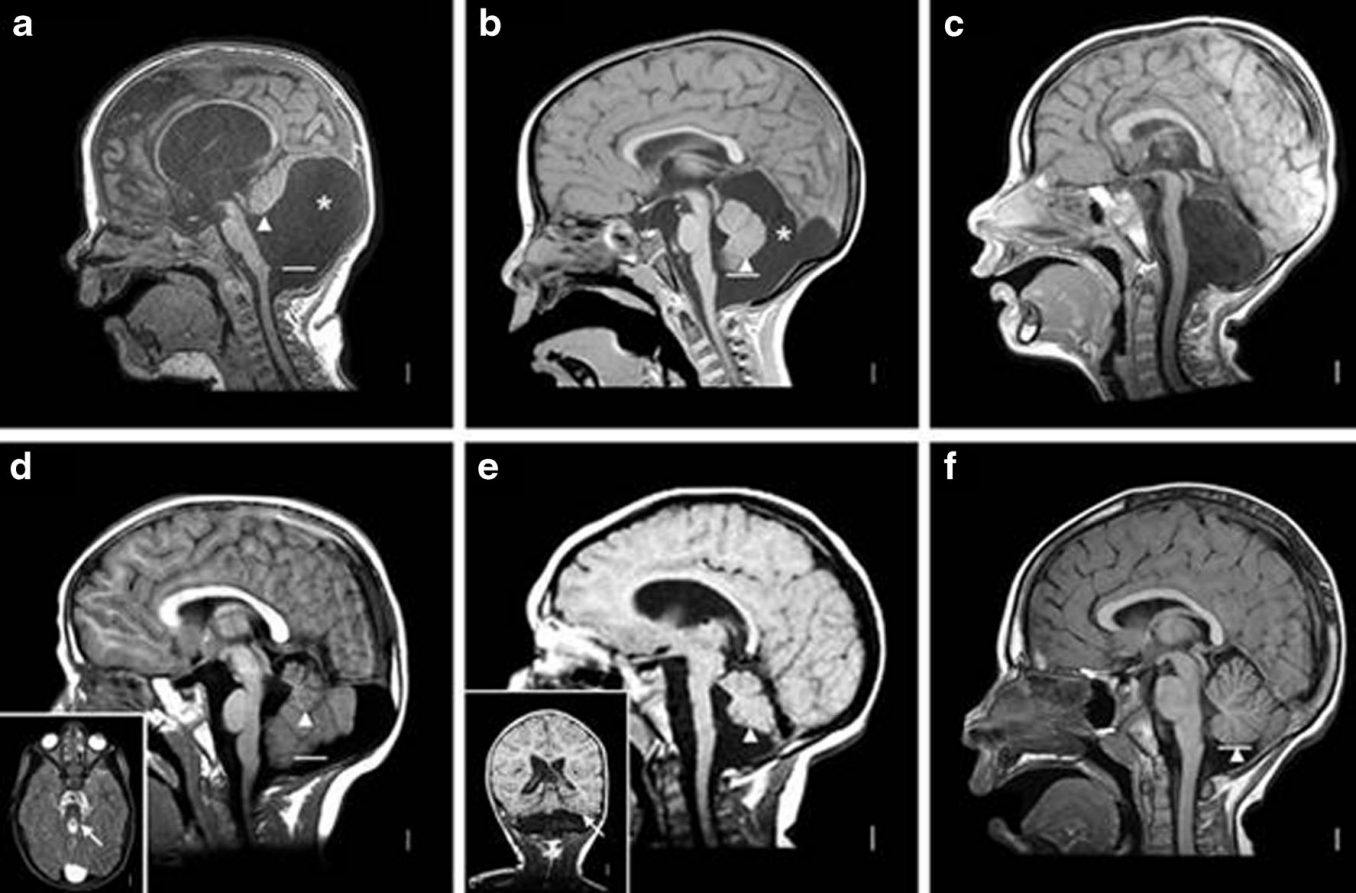
Brain imaging in mid-hindbrain malformations. T1-weighted midline sagittal magnetic resonance images show the key features of classic DWM (**a**), cerebellar vermis hypoplasia with mega-cisterns magna (**b**), complete cerebellar agenesis (**c**), molar tooth malformation seen in JSRD (**d**), pontocerebellar hypoplasia (**e**), and normal (**f**). The *solid white lines* in most images mark the level of the obex, while the *arrowheads* point to the lower edge of the vermis (both landmarks are absent in **c**). The *asterisk* denotes an enlarged posterior fossa. In (**a**), the vermis is small and rotated far upwards, the fourth ventricle is enlarged into a cyst-like structure, and the posterior fossa is greatly enlarged causing an elevated tentorium. In (**b**), the vermis is small but located in the anatomic position, but the posterior fossa is again greatly enlarged. A posterior extension of the cyst appears to scallop the inner table of the skull. In (**c**), the brainstem is thin without any landmarks other than the tectum, and no cerebellum is seen. In (**d**), the vermis is very small but located in the correct anatomic position, with portions of the cerebellar hemispheres seen beneath. The *inset* shows the associated “molar tooth” sign (*arrow*). In (**e**), the brainstem is thin but the obex can just be seen, and the vermis is moderately small. The even more “pancake-like” flattening of the hemispheres is shown in the *inset* (*arrow*)

Dandy Walker Malformation (DWM) is the most common human cerebellar malformation with an estimated incidence of 1/3000 live births [365, 366]. DWM is an imaging diagnosis characterized by an enlarged posterior fossa, cerebellar vermis hypoplasia, and an enlarged fourth ventricle (Fig. 14a). DWM can occur in association with agenesis of the corpus callosum, but more often occurs as an isolated finding on MRI scans. DWM clinical features are variable. Patients may exhibit symptoms ranging from intellectual disability to autism or they may be completely unaware of any deficits until diagnosed as adults for unrelated reasons [367–369]. The genetic causes of DWM remain largely unknown. However, recent studies indicate that deletions in *FOXC1* and *ZIC1*/*4* are responsible for a small subset of DWM cases [370, 371]. Prenatal cerebellar hemorrhage however can also cause DWM [372], which may also be associated with genetic risk factors; however, these have yet to be determined. Research in animal models has led to the hypothesis that disruptions of posterior fossa signaling from the mesenchyme surrounding the brain to the underlying embryonic cerebellum are key. Signaling disruptions cause dramatic reductions in cerebellar anlage neuronal progenitor proliferation, as well as abnormal migration of both RL- and VZ-derived cells. This ultimately leads to foliation and lamination defects [370, 373].

Joubert syndrome and related disorders (JSRD) is a group of disorders with an incidence of 1 in 80,000–100,000 live births [374, 375]. JSRD is characterized by cerebellar vermis hypoplasia, thick and abnormally oriented superior cerebellar peduncles, and a deep interpeduncular fossa, all of which give it the pathognomonic Molar Tooth Sign (MTS) seen in axial brain scan images ([376], Fig. 14d). Patients with JSRD exhibit variable neurological symptoms such as ataxia, developmental delay, abnormal eye movements, and altered breathing patterns. To date, ~23 genes have been identified as causative for JSRD [377, 378]. Most have been linked to the primary cilia and its function, bringing JSRD under the umbrella of a highly heterogeneous group of disorders called ciliopathies. Studies in animal models as well as human fetal tissue from JSRD patients indicate reduced GC proliferation suggesting impaired SHH signaling ([290, 291, 379]: see “Extrinsic Regulators of Cerebellar Development: The Role of Sonic Hedgehog” section). Additionally, the primary cilia also plays a role in the mediation of signaling pathways involving WNT and platelet-derived growth factor which can impact cerebellar anlage fusion earlier in fetal development [380, 381].

Cerebellar hypoplasia (CH) refers to underdevelopment of the cerebellum. This category of cerebellar malformation is distinct from DWM, as it does not involve a concomitant enlargement of the posterior fossa. CH is also an extremely heterogeneous group of disorders, and often, other CNS abnormalities are observed, including lissencephaly, microcephaly, and cortical heterotopia. CH may be unilateral, global, vermian, or ponto-cerebellar, where in addition to the cerebellum, the volume of the pons is also reduced likely reflecting the common developmental origin of GCs and pontine nuclei neurons in the cerebellar RL ([20, 382], Fig. 14b, e). In contrast to DWM, almost all individuals exhibit cognitive and motor impairments. Several genes have been associated with CH including mutations in *CASK*, *DAB1*, *OPHN1*, *RELN*, *CHD7*, several tubulin genes, and several *TSEN* genes [209, 383–392]. Each causes developmental defects in a multitude of cerebellar developmental programs, including progenitor proliferation and neuronal migration and even developmental cell survival. Notably, CH can also occur due to a variety of non-genetic causes such as perinatal cytomegalovirus infection and perinatal exposure to alcohol and drugs such as cocaine [393–397].

Cerebellar agenesis is an extremely rare anomaly distinguished by a complete or near-complete absence of the cerebellum ([398]; Fig. 14c). Individuals show a number of neurological deficits particularly related to movement and speech, but can be otherwise surprisingly unaffected [399]. Homozygous mutations in *PTF1A* have been associated cerebellar agenesis in humans [400]. In mice, *Ptf1a* is required for the generation of all VZ-derived GABAergic cerebellar neurons. Failure to generate these neurons means that RL-derived cells have no trophic support and these too are therefore lost, resulting in cerebellar agenesis in neonates [34]. Fetal hemorrhages that completely disrupt the early cerebellar anlage have also been predicted to cause cerebellar agenesis [401].

Recent developments in neuropathology and neuroimaging have tremendously improved the diagnosis of developmental disorders of the cerebellum. Several genes responsible for these heterogeneous malformations have been identified and animal models have revealed novel developmental mechanisms of interest to both clinical and basic science. A deeper appreciation of the cellular and signaling mechanisms responsible for these malformations will enable improved diagnosis and potential treatment of these disorders.

### The Role of Thyroid Hormone in Cerebellar Development (N. Koibuchi)

The importance of T3 and T4 in brain development has been well documented [402, 403]. Deficiency of TH during fetal and early postnatal period results in severe mental retardation, known as cretinism in humans [404]. Since there is a distinct “critical period” of TH action in brain development, replacement of TH should be started as early as possible to prevent irreversible neurological disorders.

T4 enters the brain through the blood–brain barrier (BBB) more easily than T3, an active form of TH [405]. After crossing the BBB, T4 is taken up by astrocytes and deiodinated to produce T3 by type 2 iodothyronine deiodinase [406]. T3 is then transferred to neurons or oligodendrocytes, possibly via monocarboxylate transporter 8 [407]. TH effects are mainly exerted through the nuclear TH receptor (TR; TRα1, TRβ1, and TRβ2), a ligand-dependent transcription factor [408]. TR binds to specific DNA enhancer sequences known as the TH-response elements located in the promoter region of target genes [408]. The rodent cerebellum is a good model to investigate the TH action. This is partly because the rodent cerebellar development occurs largely postnatally, allowing cerebellar TH status to be precisely manipulated at various developmental stages [402].

Perinatal hypothyroidism dramatically affects morphogenesis [402, 403]: the growth, dendrite arborization, and dendrite spine formation of PCs are all markedly decreased; synaptogenesis between PCs and PFs is dramatically repressed; the disappearance of the EGL is postponed as a result of the delayed proliferation and migration of GCs into the GL [409]. TRs are expressed in the most subsets of cells in the developing cerebellum [410]. TRα1 is abundant in GCs, whereas TRβ1 is mainly expressed in PCs. The expression of many cerebellar genes is altered by perinatal hypothyroidism [403]. Representative TH-responsive genes in the cerebellum include neurotrophins such as nerve growth factor, BDNF, NT3, and NT-4/5, and receptors such as the inositol trisphosphate 3 receptor, and retinoic acid receptor-related orphan receptor α, hairless, and myelin basic protein [411, 412]. The expression of many of these genes is regulated by TH only during a limited period of development. (It should be noted, however, that although TH action in the brain is greater during development, TR levels are greater in the adult brain: [413].) Thus, TH sensitivity may be controlled by other unknown epigenetic mechanisms such as DNA methylation and histone modification.

Various animal models have been used to study TH in cerebellar development [414]. Interestingly, TRα knock-out mice, TRβ knock-out mice and TRα/TRβ double knock-out mice do not display obvious cerebellar defects, suggesting that most of the consequences of congenital hypothyroidism in the brain are due to the detrimental activity of unliganded TR. This hypothesis is supported by studies of transgenic animals expressing mutant TR, which show severe neurodevelopmental defects [415, 416].

**Fig. 15:**
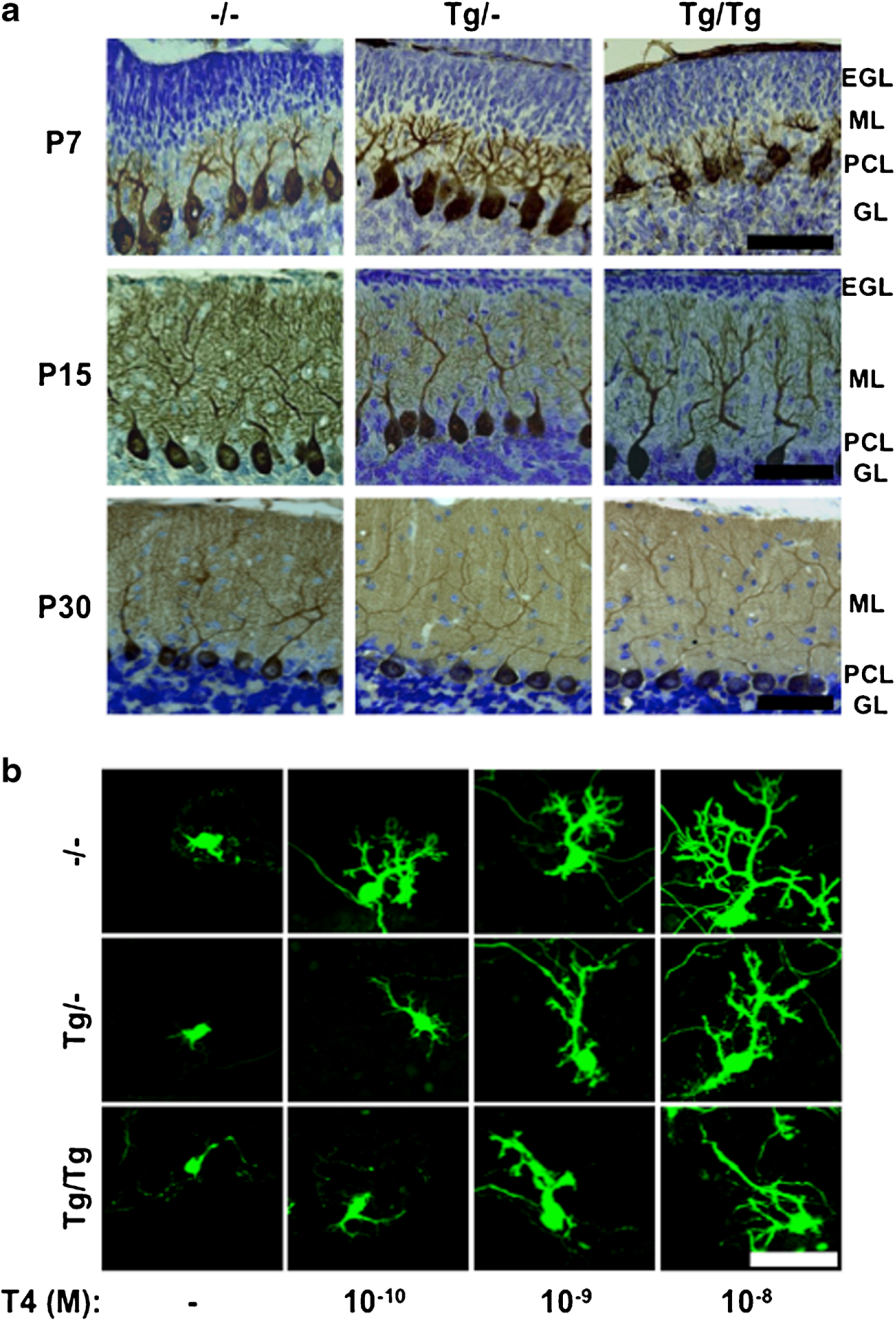
Morphological alterations in the postnatal cerebellum by mutant TR in the Purkinje cell. Mutant human TR, which inhibits normal TR action, is expressed in PCs by using the L7/Pcp2 promoter. **a** Sagittal sections of the cerebellar vermis at P7, P15, and P30 were stained with mouse anti-calbindin-D28K (1:1000) and cresyl violet. Note that the EGL is seen in He and Ho mice on P15. *EGL* external granular cell layer, *ML* molecular layer, *PCL* Purkinje cell layer, *GL* granular layer, *Tg/-* heterozygote, *Tg/Tg* homozygote, −/− wild-type. *Bar*, 50 μm. **b** Changes in PC dendrite arborization in primary cerebellar culture. After 17 days in vitro, with or without T4, the cells were fixed, and immunocytochemistry was carried out using the anti-calbindin antibody to visualize PCs. *Bars*, 50 μm. *Tg/-* heterozygote, *Tg/Tg* homozygote, −/− wild-type. Adapted with permission from [418]. *EGL* external granular layer, *ML* molecular layer, *PCL* Purkinje cell layer, *GL* granular layer

Although these animal models have contributed greatly to our understanding on the role of TR in cerebellar development, these may not sufficiently address the mechanisms of direct TH action. Since TH acts not only in the brain but also in the peripheral organs, brain development may be affected by peripheral metabolic changes. Thus, cell or organ-specific inhibition/activation of TH action is required. For such purpose, Fauquier et al. [417] used a L7/Pcp2 promoter to generate transgenic mice that express a mutant TRα1 specifically in PCs after P8. Probably because the timing of transgene expression is slightly after the critical period, this mouse showed only limited alterations in cerebellar morphogenesis. On the other hand, by Ptf1a-Cre recombination, mutant TRα1 was expressed in PCs and GABAergic INs from prenatal stages, showing the alteration of PC morphogenesis [417]. We have also generated a transgenic mouse using mutant human TRβ1 with *L7*/*Pcp2* promoter (Fig. 15; [418]). Expression of mutant TRβ1 was observed as early as P2. This mouse showed decreased PC dendritic arborization and lower levels of expression of TH-regulated genes in PCs. To our surprise, GC migration was also retarded and the expression of TH-regulated genes in GCs and oligodendrocytes was also decreased. As a possible consequence, this mouse shows cerebellar ataxia. These studies indicate that TH may mainly act through TR in the PC to regulate the whole cerebellar development. Additional factors that transmit TH signaling in PCs to other subsets of cells are required. Furthermore, although it is usually considered that the critical period for TH action in the rodent cerebellum is the first two postnatal weeks, the actual critical period may be earlier. Disruption of TH action by environmental factors during the critical period may produce adverse effects [419].

### Abnormal Purkinje Cell Development and Cerebellar Ataxia (E.B.E. Becker)

The cerebellar ataxias comprise a heterogeneous group of neurological disorders characterized by gait disturbances, motor incoordination and imbalance, dysarthria, and oculomotor deficits [420, 421]. The etiology of cerebellar ataxia is complex and includes acquired causes as well as a steadily growing number of inherited conditions [421–423]. The genetic ataxias are usually progressive. For many of these disorders, pathologic changes in PCs and a substantial loss of these neurons resulting in cerebellar atrophy are thought to cause the symptoms of the disease. However, accumulating evidence from cell- and animal-based models of cerebellar ataxia suggest that abnormal PC development and related early changes in PC physiology might contribute to the disease, thus challenging our view of cerebellar ataxias as pure neurodegenerative disorders. Here, I briefly review the emerging concept that PC developmental abnormalities might be contributing to disease pathogenesis in cerebellar ataxia.

Spinocerebellar ataxia type 1 (SCA1) is caused by a CAG repeat expansion in the Ataxin1 (*ATXN1*) gene and is one of the most intensely studied dominant ataxias. Numerous mouse and other animal models have been generated for SCA1 that recapitulate different aspects of the human disease. For example, both transgenic mice overexpressing expanded *ATXN1*[*82Q*] as well as knock-in mice (*SCA1* ^154Q/2Q^) exhibit motor impairments and PC degeneration [424]. Interestingly, transgenic mice in which expression of the expanded transgene is delayed until well after the cerebellum has matured display a much reduced disease phenotype, suggesting that mutant ATXN1 interacts with a pathway involved in PC development [425]. Indeed, the same study demonstrated a key interaction of ATXN1 with retinoic acid-related orphan nuclear receptor α (RORα), a transcription factor critical for cerebellar development. Moreover, RORα expression levels were found to be reduced in the *ATXN1*[*82Q*] model [425]. Taken together, the results of this landmark study provided the first functional genetic evidence that compromising PC development contributes to the severity of neurodegeneration. Subsequent studies have shown that SCA1 transgenic mice display abnormalities in PC development, including a reduction of CF translocation along the developing dendritic tree and decreased pruning of CF terminals from the PC soma [426, 427]. Similarly, profound impairments in PC dendritogenesis, spine development, and synaptogenesis have been described in the *staggerer* mouse [428, 429], which harbors an autosomal recessive mutation in the *Rora* gene encoding RORα, and is viewed by some as an extreme model of SCA1 [429].

Other studies have demonstrated similar PC developmental abnormalities in different mouse models of degenerative cerebellar ataxias. A PC-specific transgenic mouse model that expresses a truncated form of expanded human Ataxin-3, the disease protein causing SCA3, displays disarrangement of PCs and poor PC dendritic arborization [430, 431]. As described above for SCA1, the PCs in this SCA3 model express decreased levels of the developmental transcription factor RORα [431], suggesting a potential molecular link between the observed developmental abnormalities in both models.

Impaired dendritic arborization of PCs as well as abnormal spine morphogenesis have also been described in mice lacking *β*-III spectrin, a model of SCA5 [432]. Similarly, cultured PCs overexpressing PKCγ with SCA14-causing mutations after adenoviral infection display a decreased dendritic arbor as well as decreased spine density [433]. In vivo lentiviral-mediated expression of mutant PKCγ in PCs led to impaired pruning of CF synapses from developing PCs, although no dendritic abnormalities were observed [434]. However, the recently reported transgenic SCA14 mouse model shows abnormal dendritic development of PCs both in vivo as well as in organotypic slice cultures [435].

Besides models of the human SCAs, several other genetic mouse mutants have highlighted the relationship between abnormal PC development and ataxia. For example, the ataxic *Moonwalker* (*Mwk*) mouse harbors a dominant gain-of-function mutation in the TRPC3 ion channel, resulting in adult-onset PC loss but also impairments in PC dendritic arborization during cerebellar development [436]. TRPC3 is a key player in the mGluR1 signaling pathway vital for PC function [437, 438]. Interestingly, impaired mGluR1 signaling has been demonstrated in a number of the mouse models described above including SCA1 [439, 440], SCA3 [441], and SCA5 [442] models. Future research should help to clarify whether there is a causal relationship between impaired mGluR1 signaling at PC-PF synapses and developmental PC abnormalities in these and other models of ataxia.

In summary, PC developmental abnormalities are clearly evident in a wide range of ataxic mouse mutants including models of the degenerative SCAs. The observed PC developmental defects commonly include impaired dendritic arborization, resulting in synaptic deficits affecting CF and PF connections and ultimately altering PC physiology. It will be important to better understand the underlying—likely common—molecular mechanisms by which mutations in distinct genes cause abnormal PC development and function. These could offer attractive future therapeutic targets to alleviate motor dysfunction in cerebellar ataxia.

### Deregulated Developmental Pathways in Medulloblastoma (S. Marino, T.O. Millner)

MB is the most common pediatric brain tumor and is the most common cause of pediatric death from cancer. Histologically, cases are classified into classic, nodular/desmoplastic, and large cell/anaplastic subtype [443], and prognosis is performed by combining histological subtype, clinical markers, namely, age, metastatic stage, and level of resection, as well as selected molecular markers. Morphologically, MB cells closely resemble GCs and GCPs; hence, it has been long postulated that a link exists between these tumors and the normal development of the cerebellum. Evidence gained from candidate gene approaches in mouse models and more recently “-omics” screening of large tumor series has shown deregulation of specific developmental pathways in subgroups of these tumors.

**Fig. 16:**
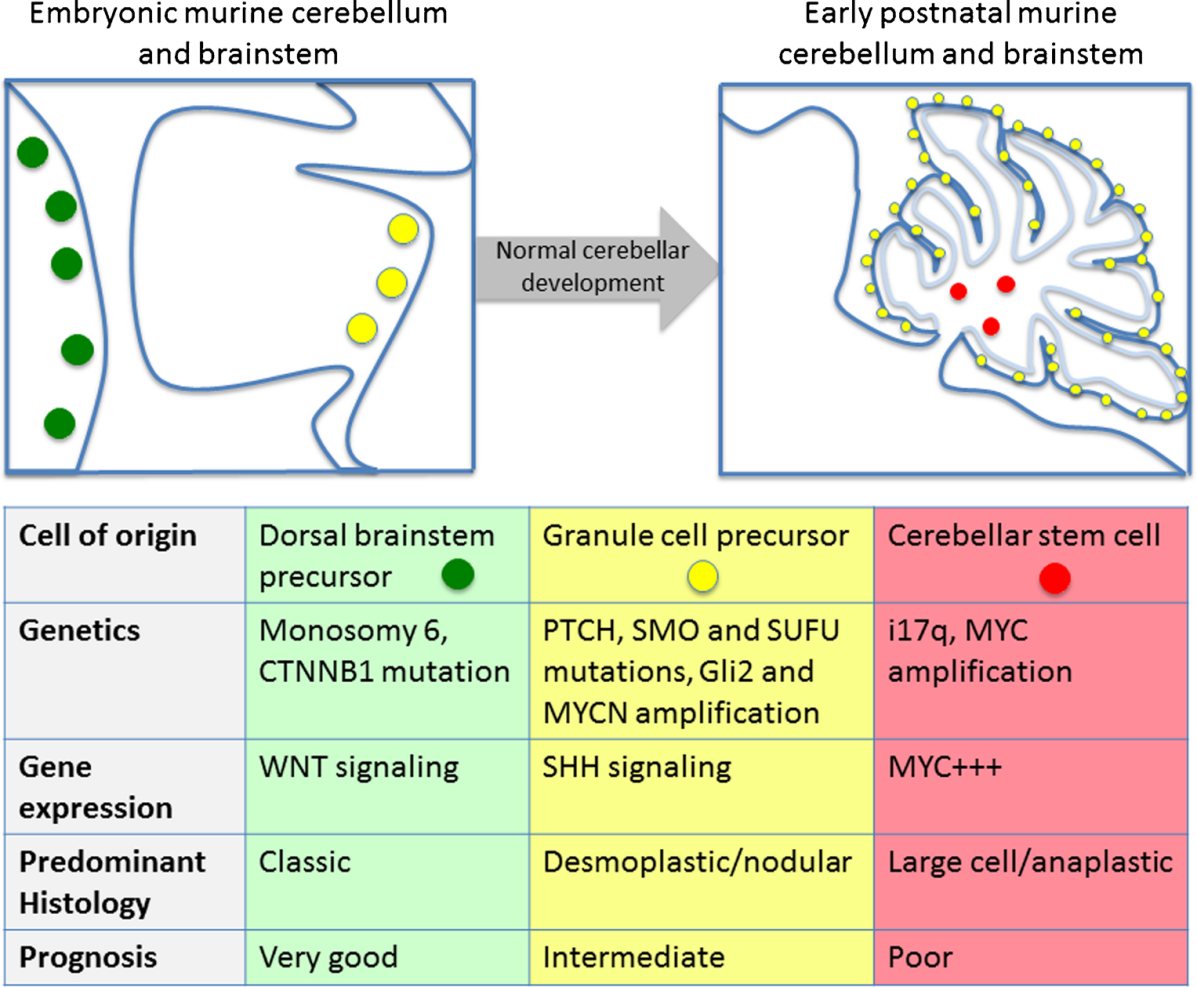
Medulloblastoma subgroups and their cells of origin. The schematic shows the embryonic and early postnatal murine cerebellum and brainstem with the spatial and temporal locations of likely cells of origin of MB subgroups (*green dots* represent dorsal brainstem precursor cells, *yellow dots* represent GCPs, *red dots* represent cerebellar stem cells). The table shows the genetics, gene expression profile, predominant histology, and prognosis of the MB subgroups for each of these cells of origin

The current consensus is that MB can be sub-classified based on genetic, epigenetic, and transcriptomic characteristics [444, 445] into four distinct subgroups (Fig. 16): WNT, SHH, Group 3, and Group 4 [445]. The WNT and SHH subgroups have been associated with constitutive activation of the WNT/β-catenin and SHH pathways, respectively, whereas Group 3 and Group 4 MBs are less well characterized. Each of these subgroups have defining demographic, clinical, genetic, and epigenetic profiles, and emerging evidence links their origin to different cerebellar progenitor cells at different developmental time points. Here, we will summarize the key features of the current molecular stratification of MB from a developmental oncobiology perspective.

#### GCPs and SHH-MB

GCPs are the main cell of origin of SHH-MB, as shown in mouse models in which *Ptch1* is conditionally inactivated in GCPs. Importantly, constitutive activation of SHH signaling induces neoplastic transformation of more undifferentiated progenitor cells only upon commitment toward a GC lineage [446]. Pre-neoplastic lesions expressed *Atho1*, a marker of the GC lineage, and showed activation of *Gli1*, *Cyclin D1*, and *MycN*, which are SHH target genes. Their gene expression profiles were more similar (differing by 34 genes) to tumor cells than GCPs (differing by 75 genes) [447]. SHH-MBs have also been shown to originate from cells located in the cochlear nuclei of the brainstem [448].

MRI studies have shown differences in the location of MB subgroups, and MB location (as well as enhancement pattern) can predict the molecular subgroup of pediatric MBs. SHH tumors are mainly detected within the cerebellar hemispheres [449–451] consistent with a GCP cell of origin. Moreover, in a series of 63 human MBs that were morphologically nodular/desmoplastic, a histological subtype likely to be typical of the SHH subgroup [452], 33 % had very close contact to the cochlear nuclei on MRI [448]. Human Shh MBs [445] have a 1:1 male-to-female ratio and a bimodal age distribution (very frequently seen in infants and adults), with a good prognosis in infants but an intermediate prognosis in other age groups. All histological nodular/desmoplastic MBs are likely SHH-MBs [452], but 50 % of SHH-MBs are of other morphology.

#### Embryonic Dorsal Brain Stem Precursors and WNT-MB

Mouse models have shown that WNT-MBs, characterized by activating mutations in the Wnt pathway effector CTNNB1, arise from cells outside the cerebellum, in the embryonic dorsal brainstem [449]. These studies also showed that the genes characterizing human WNT-MBs are more often expressed in the lower RL and embryonic dorsal brainstem than in the upper RL of the developing cerebellum. In addition, transcriptome analysis showed that the MBs arising in these mice matched human WNT-MBs.

MRI studies in patients have shown that WNT tumors are often found within the fourth ventricle (cerebellar peduncle/cerebellopontine angle cistern) and infiltrated the dorsal brainstem [449, 450], with the majority of them being continuous with the cuneate nucleus [451], a region that corresponds to the origin defined for the murine WNT-MBs described above. Human WNT-MBs [445] have a 1:1 male-to-female ratio and occur at all ages (uncommon in infants). They have a very good long-term prognosis in comparison to the other subgroups of MB (survival rate likely exceeds 90 % with current treatment). The large majority of WNT-MBs investigated so far have classic histology.

#### Cerebellar Stem Cells and Their Role as MB Cell of Origin

Cerebellar stem cells are a third likely cell of origin for human MBs. Models using *Rb* ^+^ *Tp53* mutant mice were generated where MBs developed from neural stem cells in the cerebellar white matter [453, 454]. These tumors resembled human Group 3 MBs histologically while expressing high levels of neural stem cell markers (Nestin, Sox2, and Sox9). A further model using *Myc* ^+^ *Tp53* mutant mice developed MBs from cerebellar neural stem cells and from GCPs, although the tumors that formed from GCPs lost their lineage specific markers first [455]. This model expressed high levels of neural stem cell genes and also resembled human Group 3 tumors at a histological and molecular level.

In the MRI studies to date, Group 3 and Group 4 MBs are characterized by brainstem contact with most of the tumors growing within the vermis. Most also contact both the cochlear and cuneate nuclei and always infiltrate the fourth ventricle [450, 451]. Group 3 MBs [445] are seen more frequently in males than females and almost never in adults, have a poor prognosis, and are frequently metastatic. Group 3 MBs most frequently have a classic morphology, but the Group 3 subgroup contains the majority of the large cell/anaplastic tumors.

Group 4 comprises 30 % of MBs and it is the least well characterized molecularly. At present, it is also unclear from which cells these tumors originate. This subgroup has a male-to-female ratio of 2:1 and has an intermediate prognosis, similar to the SHH subgroup [445]. Group 3 and Group 4 MBs share some molecular features: amplification of the OTX2 oncogene, which is not seen in the other subgroups, and isochromosome 17q (26 % in Group 3 and 66 % in Group 4 MBs [447]). However, there are also important differences. Group 3 MBs show high levels of MYC, and often gain of chromosome 1q and/or loss of chromosome 5q and 10q, whereas Group 4 MBs have low levels of MYC and MYCN and loss of the X chromosome in 80 % of females within the subgroup [445].

In summary, “-omics” datasets on large series of MB combined with the results of ontogeny studies performed in mouse models aiming at characterizing the cell of origin of the various subgroups together with magnetic resonance imaging (MRI) studies in patients lend additional support to the notion that MB is in fact a disease arising from deregulated cellular and molecular mechanisms involved in the development and homeostasis of the cerebellum.

## Concluding Remarks (C. Sotelo)

The goal of our consensus paper is to provide an updated view of our current knowledge on cerebellar development. The broad spectrum of the work reviewed in such a short format leaves little room for specific debate. Nevertheless, many of the concepts reported here have already reached consensus.

The seminal discovery of the “isthmic organizer” and its inductive role has led to an understanding of the molecular regionalization of the neural tube with its precise frontiers between domains expressing homeobox-containing genes. The interface between caudal GBX2 and rostral OTX2 expressing domains marks the location of the “isthmic organizer” which, through Fgf8 secretion, initiates the molecular cascade required for the specification of cerebellum.

Next come the germinal epithelia of the cerebellum. For over a century, the cerebellum was known for the dual origins of its neuronal populations: the VZ and the RL. However, the categories of neurons generated in each of these epithelia have only recently been established. Genetic fate mapping proved that glutamatergic neurons (most of the CN neurons, and all GCs and UBCs), specified by the transcription factor ATOH1, originate from the RL and migrate via the EGL. In contrast, GABAergic neurons (PCs, ML INs, Lugaro and Golgi cells, and GABAergic neurons of the CN), specified by PTF1a, originate from the VZ. Moreover, both neuroepithelia are divided into subdomains, each one specified by distinct transcription factors to generate the corresponding population of neurons.

Following specification comes neuronal migration and differentiation. Despite recent progress, the data are less complete. For example, broad areas of the early development of PCs remain obscure, even while later events such as dendritogenesis are well understood. Our knowledge on the mechanisms leading to the biochemical heterogeneity of PCs and their aggregation into longitudinal and transverse compartments has improved: there is a dichotomy among PCs, those born between E10 and E11.5 will become ZII^+^ and those born later will be ZII^−^, indicating that specification might exist among progenitors. Related to PC compartmentalization, the formation of circuit topography and the elimination of CF multiple innervation opens the vast domain of synaptogenesis and the role of functional activity in the fine-tuning of the cortical circuit’s specificity—a chapter that, at this moment, remains little explored and is an anticipated topic for future consensus papers. Similarly, the ways in which cerebellar INs and glia become integrated into the mature cerebellum are also beginning to be understood. Finally, in parallel with these events, we are now seeing how the development of the characteristic cerebellar foliation fits into the picture.

We have also updated on the role of the TH (a cerebellists’ old friend because of its multiple effects on cerebellar development) describing recent advances using transgenic animals with conditional nuclear TH receptor knock-outs targeted to PCs or/and IN that allow for the suppression of secondary effects on other neurons or peripheral organs.

Finally, our review has briefly touched upon cerebellar pathology. For example, the analysis of the role of SHH, secreted by PCs as mitogen for GC proliferation, provides a basis for better understanding of the origins of MBs, and inherited disorders of cerebellar development can now be placed in a stronger developmental context.

In conclusion, following the outline proposed by Ferdinando Rossi in his planned monograph, the data presented here provide our brief consensus of the current knowledge on cerebellar development.

## Abbreviations

BG: Bergmann glia
CH: Cerebellar hypoplasia
CN: Cerebellar nuclei
CF: Climbing fiber
DWM: Dandy Walker Malformation
E: Embryonic day (all timings are mouse unless stated otherwise)
ES: Embryonic stem cell
EGL: External granular layer
FGF: Fibroblast growth factor
GL: Granular layer
GC: Granule cell
GCP: Granule cell progenitors
HH: Hamburger and Hamilton stage
INs: Interneurons
IsO: Isthmic organizer
JSRD: Joubert syndrome and related disorders
MB: Medulloblastoma
ML: Molecular layer
VZ: Neuroepithelium of the ventricular zone of the 4th ventricle
NTZ: Nuclear transitory zone
PF: Parallel fiber
PIPs: Pax-2^+^ IN progenitors
P: Postnatal day (all timings are mouse unless stated otherwise)
PWM: Prospective white matter
PC: Purkinje cell
PCPs: Purkinje cell progenitors
RL: Rhombic lip
r1: Rhombomere 1
SCA: Spinocerebellar ataxia
(TH; L-triiodothyronine, T3; L-tetraiodothyronine, thyroxine, T4): Thyroid hormone
UBCs: Unipolar brush cells
